# Plasma Modification and Synthesis of Membrane Materials—A Mechanistic Review

**DOI:** 10.3390/membranes8030056

**Published:** 2018-08-03

**Authors:** Jingshi Wang, Xiao Chen, Rackel Reis, Zhiqiang Chen, Nick Milne, Bjorn Winther-Jensen, Lingxue Kong, Ludovic F. Dumée

**Affiliations:** 1 Institute for Frontier Materials, Deakin University, Pigdons Road, Waurn Ponds, Geelong, VIC 3216, Australia; vwq@deakin.edu.au (X.C.); zhiqiang.chen@deakin.edu.au (Z.C.); lingxue.kong@deakin.edu.au (L.K.); 2 Institute for Sustainability and Innovation, College of Engineering and Science, Victoria University, Hoppers Lane, Werribee, VIC 3030, Australia; rackel.reis@live.vu.edu.au; 3 School of Engineering, Deakin University, Pigdons Road, Waurn Ponds, Geelong, VIC 3216, Australia; n.milne@deakin.edu.au; 4 Department of Advanced Science and Engineering, Waseda University, Tokyo 169-8555, Japan; bjornwj@aoni.waseda.jp

**Keywords:** membrane surface modification, plasma texturation, plasma polymerization, wettability, free volume, plasma mechanics

## Abstract

Although commercial membranes are well established materials for water desalination and wastewater treatment, modification on commercial membranes is still necessary to deliver high-performance with enhanced flux and/or selectivity and fouling resistance. A modification method with plasma techniques has been extensively applied for high-performance membrane production. The paper presents a mechanistic review on the impact of plasma gas and polymerization, at either low pressure or atmospheric pressure on the material properties and performance of the modified membranes. At first, plasma conditions at low-pressure such as plasma power, gas or monomer flow rate, reactor pressure, and treatment duration which affect the chemical structure, surface hydrophilicity, morphology, as well as performance of the membranes have been discussed. The underlying mechanisms of plasma gas and polymerization have been highlighted. Thereafter, the recent research in plasma techniques toward membrane modification at atmospheric environment has been critically evaluated. The research focuses of future plasma-related membrane modification, and fabrication studies have been predicted to closely relate with the implementation of the atmospheric-pressure processes at the large-scale.

## 1. Introduction

Membranes have been widely applied in microfiltration (MF), ultrafiltration (UF), nanofiltration (NF), and reverse osmosis (RO) applications spanning from wastewater treatment, water recycling, desalination, to gas separation [1,2]. Membranes are, however, prone to surface fouling during operation that hinders membrane performance in terms of permeance and selectivity. To alleviate requires harsh and complex cleaning procedures associated with high energy input, which ultimately reduces their lifespan [3,4,5]. Extensive studies on membrane modification have focused on overcoming membrane fouling problems or the development of new membrane materials with high fouling resistance [2,6]. Another focus of membrane modifications was to enhance the permeance of membranes without compromising its selectivity [1,7]. 

Plasma processes have been widely used for the surface modification of polymeric membrane materials. The intrinsic properties of plasma treatments such as fast reaction time, waste-free processes, and high versatility allow them to be a promising candidate to replace conventional coating and grafting methods [8,9]. The plasma modification routes only require the use of a lower degree of ionization and are generally referred to ‘cold’ plasma processes [10]. A lower degree of ionization only delivers surface modification without damaging the bulk material of the treating substrates and thus offering smooth surface modification solutions [11]. The plasma process conditions may be fine-tuned by altering the discharge parameters, including the power, chemical properties, and flow rate of the plasma gas and precursors, as well as treatment durations [12,13,14]. Plasma gas processes, on the one hand, often referred to as plasma functionalization, have been primarily applied for surface modification and chemical implantation induced by non-polymerizing gases [15]. Plasma polymerization, on the other hand, has used plasma discharge to activate and polymerize organic precursors to deposit a layer of plasma polymerized materials [16].

A number of publications have reviewed membrane modification studies using plasma-related techniques. The applications of microwave powered plasma-associated processes have also been summarized, including plasma gas, polymerization, and plasma-induced polymerization for membrane modification, with limited information related to the performance of the modified membranes [17,18], while others highlighted that properties and performance of the modified membrane upon low-pressure plasma processes based on the case studies published between 2000 and 2010 [19]. The benefits from modified polymeric membranes upon plasma gas and polymerization on antifouling properties were reported [20]. Surface hydrophilicity enhancement routes induced by plasma processes were highlighted in a detailed review of current surface modification practices for water purification membranes [21]. In addition, the reviews related with pure plasma physics and chemistry have also been reported for low- and atmospheric-pressure processes [22,23]. 

In the past decade, studies have not only focused on the impact of plasma on the material properties of the modified membranes, but also tried to correlate the changes of material properties with membrane performance. This review aims to provide a mechanistic discussion on the impact of plasma processes on the material proprieties including surface wettability, morphology, roughness, and surface charge. Moreover, the performance change of the plasma modified membranes is then critically reviewed with regard to changes in the material property. Thereafter, the mechanisms of the chemical reactions occurring in the plasma treatments are provided at the end of each section.

This review will be focused on cold plasma applications carried out under either low- or atmospheric-pressure conditions [24]. It should be highlighted that plasma-induced/activated polymerization processes will not be reviewed in this review, as the underlying mechanism of this type of process falls into the combination of plasma gas and chemical polymerization which does not fit the scope of this review.

## 2. Low-Pressure Plasma Processes

### 2.1. Plasma Gas Treatments of Membranes

Plasma processes using non-polymerizable gases modify polymer and membrane materials by inducing plasma etching [25,26,27,28,29,30]. During this process, the membrane surface undergoes a bombardment with electrons and ions from the plasma-phase. The kinetic energy of these species can be in the range of several electron volts and are thereby able to break any bond in polymeric materials, which thereby is etched as low molecular fragments are escaping the surface of the material. A part of the etching reaction is the formation of radicals in the polymer surface as a result of the bond breaking. When inert gasses are used (e.g., Ar or He) these radicals can only either re-react with polymer fragments or typically react with oxygen when the polymer is exposed to air after the treatment. In the case of reactive gasses (O_2_, CO_2_, H_2_O, NH_3_, etc.), the radicals formed on the polymer surface can, in addition, react with these gasses or with fragments of them created in the plasma phase [22,31]. The plasma generated from oxidative, reductive, or inert gases and gas mixtures have been widely applied to modify a number of polymeric membranes.

Investigated membrane materials include microfiltration (MF) poly(carbonate) (PC), poly(ethylene terephthalate) (PET) [32], poly(tetrafluoroethylene) (PTFE) [33], and ultrafiltration (UF) PET [34], poly(propylene) (PP) [35], poly(sulfone) (PSf) [27,36], and poly(ethersulfone) PES [37,38], and poly(vinylidene fluoride) PVDF [39]. Recently plasma gases were used to alter reverse osmosis (RO) poly(amide) PA thin film composite (TFC) membranes [7,29].

In this section, the impact of specific plasma gas processes on membrane material properties such as surface wettability, chemical composition, morphology and roughness, and membrane performance regarding water flux and fouling resistance will be described. Particularly, the mechanisms of plasma gas treatments will be elucidated at the end of this section.

#### 2.1.1. Inert Gas Plasma—Argon and Helium Plasma

Argon and Helium are not able to directly introduce new functional groups onto the surface of membrane materials but can induce both functionalizations and cross-linking of the outmost layers of the polymeric materials [40]. Inert gases are also commonly used in combination with other gases or monomer precursors to help to produce homogeneous plasma discharges. Ar gas is typically used as a carrier or a support, and has previously been used along with ammonia plasma, as discussed in Section 2.1.3. Reductive gas plasma—ammonia (NH_3_), as well as during plasma polymerization processes as further presented in Section 2.2. Low-pressure plasma polymerization treatments toward to surface modification [24,41]. This section only presents direct usage of inert gases to modify membranes.

Inert gas plasma has been applied to produce hydrophilic membrane surfaces by texture alteration or by etching specific functional groups from the polymer surface. One of the early plasma studies compared the impact of O_2,_ and Ar plasma on the laboratory synthesized RO PA TFC membranes (Entry 5 in Table 1) [40] and highlighted that Ar plasma is less efficient than O_2_ plasma in producing hydrophilic membrane surfaces. For example, the WCA of the RO PA-treated with 6 min O_2_ and Ar plasma decreased by 43% and 10% from 77°, respectively [40]. The insignificant hydrophilicity enhancement induced by Ar plasma was likely due to the addition of the polar functional groups mainly occurred after the treatment when the polymer was exposed to oxygen from air. Consequently, the flux of the modified membranes changed accordingly to the macroscopic hydrophilicity variations observed. The water permeability of the modified RP PA was more than 2.5 times higher than that of its control membranes (0.49 m^3^/m^2^/day) after 3 min of O_2_ plasma at 99 W. In contrast, Ar plasma facilitated a 4% increase in flux for RO PA membranes treated in the same plasma discharge conditions [40]. 

Furthermore, two recent studies highlighted the impact of Ar and He plasma power and duration on RO PA (BW 30 TFC, DOW) [7,29]. In accordance with the other inert gas plasma treatments, Ar and He plasma have demonstrated their ability to enhance the hydrophilicity of the RO PA surface. After a 5 min plasma treatment at 10 W, the WCA of the modified PA declined by 60% from 60° (PA1 control) and the WCA of the second PA control (PA2) dropped 78% from 47° [7,29]. Consequently, the water flux of the RO PA modified by Ar and He plasma increased by 22% and 66% in comparison with the pristine PA1 (44.9 ± 1.2 L m^−2^ h^−1^) and PA2 (30 L m^−2^ h^−1^), respectively [7,29]. However, the flux loss dramatically surged after increasing plasma power and duration. The water flux of the Ar plasma modified PA1 was reduced by 76% compared to that of the pristine PA1 after increasing the plasma power to 80 W and the duration to 30 min [7]. As a result of a higher plasma power over the course of longer treatments, the flux reduction was likely attributed to the deposition of sputtered fragments etched and vaporized from the surface thus leading to a smoother surface with lower surface porosity [29]. This treatment may have led to partial melting/softening of the surfaces leading to an apparent polishing of the initially present rough surface. In case of the He plasma, the water flux of PA2 increased by 25% after the plasma power increased from 10 to 80 W, suggesting that the effect of plasma etching outperformed the re-deposition mechanisms for shorter plasma discharge durations. The weaker etching impact compared to Ar plasma is because that He is significantly lighter than Ar [24]. 

The impact of the plasma power on the membrane selectivity was also revealed by studying the salt rejection of a 2000 ppm NaCl solution. The salt rejection of the PA membranes modified by a 5 min Ar and He plasma at 10 W remained above 96% [7,29]. However, the salt rejection of the RO PA modified by Ar and He plasma was altered differently at higher power discharge. For instance, the RO PA modified by Ar plasma (80 W and 30 min duration) was found only to reject 5% of NaCl, whereas salt rejection for RO PA modified by He plasma (80 W and 5 min duration) was maintained at 98% [7,29]. Both flux and salt rejection results suggested that He plasma is a relatively mild modification process compared to Ar plasma, since there was no significant decline in membrane permeability and selectivity after increasing plasma power from 10 to 80 W. In contrast, although the decline of water flux was likely due to the highly dense structure of the modified RO PA, the structure of the modified PA was not as dense as its control as it lost its original selectivity against NaCl. 

#### 2.1.2. Oxidative Gases Plasma Including Oxygen/Carbon Dioxide/Water Vapor

Plasma of oxidative gases is mainly used to increase the hydrophilicity or wettability of membrane materials by introducing polar functionalities to the membranes [32]. In addition to chemical changes, oxidative plasma can also alter the physical structure of the membranes with pore enlargement in microporous membranes, leading to improved water flux.

##### Oxygen Gas Plasma

Upon O_2_ plasma treatment, the polymer membrane is expected to be functionalized with oxygen-rich polar functional groups such as hydroxyl, carbonyl or carboxyl. Laboratory synthesized, UF PP membranes, were treated with O_2_ plasma (30 W, RF power) to produce oxygen-enriched surfaces (Entry 3 in Table 1) [25]. The change in membrane properties was studied as a function of plasma treatment duration [25]. X-ray photoelectron spectroscopy (XPS) results revealed that atomic ratio of O/C increased from 2.8% to 23.3% after 9 min plasma treatment, compared to the untreated pristine PP membranes. The static water contact angle (WCA) of the modified PP declined sharply by ~30% from 128° to below 90° after 30 s O_2_ plasma, and continuingly reduced to 72° after 9 min treatment [25]. These analyses suggested that the enhanced hydrophilicity was attributed to the formation of oxygen-rich functional groups across the modified PP. Such correlation was also obtained in the studies of a commercial PP membrane (osmonics) (Entry 4 in Table 1) and a laboratory synthesized RO PA membrane (Entry 5 in Table 1) upon O_2_ plasma treatments. In comparison to their pristine membrane controls, the O/C atom ratio raised from 0.014 to 0.49 and from 0.15 to 0.68 for the commercial PP and synthesized PA after 5 and 6 min O_2_ plasma treatments, respectively [26,40]. The WCA of the PP and PA decreased by 85% and 43% from its controls 135° and 77° after 5 and 6 min treatments, respectively [26,40]. Especially, there was a 9% drop in contact angle after 2 min of treatment for PA membranes, indicating that membrane surface became more hydrophilic along with plasma treatment duration.

**Figure 1:**
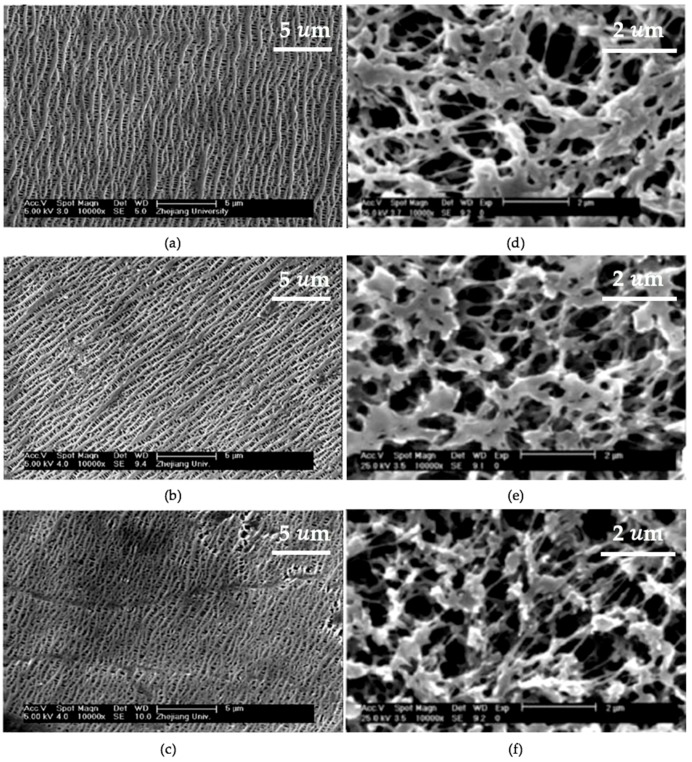
SEM images of the UF PP membranes (**a**) pristine, (**b**) 4 min, (**c**) 9 min, and MF PP membranes (**d**) pristine, (**e**) 3 min, (**f**) 5 min treatment with O_2_ plasma. The length of the scale bars in SEM images (**d**–**f**) is 2 µm. Reprinted from [25], Copyright (2018), with permission from John Wiley and Sons, and from [26], Copyright (2018), with permission from Elsevier.

The morphology change of the membranes upon O_2_ plasma was probed by scanning electron microscope (SEM). The modified PP membranes from Entry 5 in Table 1 showed that pore size increased with plasma duration until 4 min, but became smaller after increasing plasma duration to 9 min, as shown in Figure 1a–c [25]. The SEM images suggested that plasma treatment was able to etch membrane surfaces and led to the enlargement of the pores size, and the etched surfaces or enlarged pores can be restored as the duration of the plasma treatment increased. Entry 5 stated that increasing plasma duration provided enough time which allowed the deposition of ionized fragments from the etched surface took place in the plasma chamber and led to the refill of the etched membrane surfaces [25]. In contrast, plasma etching was the main mechanisms in Entry 6 (Table 1). Figure 1d–f showed that the pore size became larger as plasma treatment duration increased [26]. The morphology change, in this case, is likely due to the natural structure of the base membrane. The deposition of the sputtered fragments onto MF PP with 0.22 µm mean pore size was expected to make a less significant change in the morphology of MF PP, compared to that of the UF PP (0.10 µm mean pore size) in Entry 3. 

Furthermore, the performance of the modified membranes was reported to change in line with the morphological changes. Entry 3 (Table 1) evaluated the membrane performance based on the relative flux (%) which is the fraction of the water flux of the modified membranes over that of the pristine ones [25]. The relative water flux arose 130% after the first minute of O_2_ plasma. However, the relative water flux of the modified PP membranes (Entry 3 in Table 1) increased by 115% after 4 min treatment, which is 15% lower than the PP treated for 1 min alone [25]. The water flux results of Entry 3 supported the change in the morphology of the UF PP membranes and the deposition mechanisms. Increasing plasma duration from 1 to 4 min resulted in less flux rise, which is due to the refill of the etched membrane surface and pore. In the case of Entry 4 (Table 1), water flux was reported to be continuously increasing as plasma duration increased. The water fluxes of the modified PP increased 18% after 3 min of treatment, and by 99% after 5 min of treatment, benchmarked against the pristine PP, offering a nominal flux of 220 L m^−2^ h^−1^ [26]. The flux results of Entry 4 were in agreement with the morphology change of the MF PP. The increasing water flux was a result of enlarging pore size as plasma duration increased, which highlights the impact of the plasma mechanisms on the material properties and performance of the modified membranes.

##### Carbon Dioxide Gas Plasma

Plasma gases generated from CO_2_ were used to enhance the hydrophilicity of UF PSf (US Filter Inc. 99 kDa) and PES (Millipore Corporation) membranes (Entries 6 and 7 in Table 1) [27,28]. The impact of the plasma powers and treatment durations on the material properties and performance of PSf and PES was highlighted. In the case of the PES-treated for a 30 s CO_2_ plasma, the water drop disappeared within 75 s from the surface treated at 20 W from 66°, but within 25 s from the sample treated at 35 W [28]. For the PSf membranes, however, the impact of the plasma duration was studied at a fixed plasma power level (10 W). The WCA of the PSf treated for 180 s dropped from 94° to 0°, whereas the WCA of the PSf treated was 15° after a 30 s treatment [27]. An increasing amount of oxygen-rich polar functional groups were formed under higher plasma power levels and longer treatment duration conditions, contributed to the hydrophilicity enhancement of the modified membranes. 

Furthermore, although the oxygen-rich groups were developed on top of the modified PSf and PES membranes, the quantity of oxygen did not vary extensively between the different plasma conditions. The oxygen content of the modified PES was approximately 10% higher than that of the pristine PES (20% O) for all plasma treatment conditions (20–35 W for 0.5 to 15 min) [28]. Similarly, the oxygen content of the pristine PSf (12.8%) raised to ca. 30% and remained at this level albeit the plasma power and treatment time had increased from 5 to 10 W and 10 to 300 s, respectively [27]. In addition, these plasma conditions offered an intriguing impact on the incorporation of carbon–oxygen functional groups onto the polymer membranes. For the PES membranes treated at 20 W, 5.4% of the carbonyl group (C=O) and 9.4% of the ester or carboxylic acid group (COO–/–COOH), which was not detected at the surface of the pristine PES, appeared for plasma treatments longer than 2 min [28]. For the PSf membranes treated at a plasma power of 5 W, the content of C=O increased from 2.8% to 8.3% as treatment duration increased from 10 to 180 s, and the COO–/–COOH groups were only formed after 180 s of treatment [27]. Longer treatment durations led to higher densities of oxygen-rich polar functional groups and consequently, a hydrophilicity enhancement. 

Water flux was measured to directly represent the performance of the PSf upon CO_2_ plasma in Entry 6 (Table 1), whereas the performance of the PES membranes after plasma treatment was not reported in Entry 7, solely focused on morphological and chemical changes (Table 1). The water flux across the PSf treated at 10 W for 60 s raised to ~412 L m^−2^ h^−1^, which was over two times higher than that of the PSf control (175 L m^−2^ h^−1^) [27]. The impact of different plasma durations was studied based on the protein adsorption rate and the level of flux recovery after cleaning. The protein adsorption rate was calculated by weighting the deposition of protein after protein fouling tests. The flux recovery rate was defined as the percentage of flux measured after water cleaning over the initial flux for pure water. The protein adsorption rate of the 60 s treated PSf was approximately 36 µg cm^−1^ which was 12 µg cm^−1^ lower than that of the 30 s treated ones [27]. Furthermore, the 60 s treated PSf achieved 98% of flux recovery which as 17% higher than that of the 30 s treated PSf membranes [27]. Since SEM images showed no damage or crack across the surface of the modified PSf, the hydrophilicity enhancement was likely the underlying cause of the increased water flux upon CO_2_ plasma, and the hydrophilicity properties of the modified membranes increased with plasma duration.

##### Water Vapor Plasma

Water vapor plasma was used to enhance the hydrophilicity of UF PSf [42], PES, and PE [37], MF PC and PET (Sterlitech Corporation) [32], and RO PA TFC (Dow Filmtec Corporation) [29] (Entries 8–11 in Table 1).

The contact angle measurement was used to analyze the hydrophilicity changes across the modified membranes. On the one hand, UF PSf and PES transited from hydrophobic into hydrophilic surfaces upon H_2_O plasma, as the water droplet disappeared within 2 s from the modified surfaces of the PSf and PES with the original WCA of 90° and 70°, respectively [37,42]. On the other hand, water drop disappeared within 25 s of being applied to the PE (123°) [37], likely due to the inert nature of PE material in compare to PSf and PES which contains polar sulfonyl functional groups. Furthermore, the WCA of the MF PC and PET decreased from 97° to 35° and 54° to 25°, respectively [32]. The surface of the MF PC and PET (Entry 9 in Table 1) was not as wettable as that of the UF PES and PE membranes (Entry 10 in Table 1) upon H_2_O plasma under the same treatment conditions, although the PC and PET both contained carbonate and carbonyl functional groups. These studies highlighted that the structure of the treated surfaces other than plasma parameters had a remarkable influence on the physicochemical properties of the products [11].

The XPS analysis showed that the hydrophilicity enhancement was caused by the oxygen-enrichment upon H_2_O plasma. An increase of approximately 8–10% in oxygen content atop of the PES and PSf, and 24% atop of the PE which originally contained no oxygen compounds in its chemical structure, was found [37,42]. For these MF membranes, the O/C ratio increased by 103% and 72% for PC and PET, respectively [32]. Carbon and oxygen associated functional groups were estimated in the form of ketone or aldehyde, carboxylic acid or ester across the membrane surface upon H_2_O plasma based on the deconvolution analysis of C1s spectra [32]. The impact of the plasma gas on the topography of the modified membranes in term of surface roughness were firstly reported across RO PA TFC (Entry 11 in Table 1). After 2 min of plasma treatment, the surface roughness of the pristine membranes treated at 10 and 80 W reduced by approximately 8% and 43% from the 63 nm control, respectively [29]. The significant reduction in surface roughness at 80 W was likely resulted from smoothing effects induced at higher plasma power [29]. In addition to the etching effect, the study also speculated that the deposition of sputtered fragments from gas vapor and the etched surface led to a smooth surface with low porosity [29]. 

Furthermore, the impact of H_2_O plasma on the membrane performance was reported regarding water flux and selectivity. For example, the performance change of the RO PA TFC was studied at a different power level (10 or 80 W) and treatment duration (1–5 min) (Entry 11 in Table 1) [29]. At 10 W, there was no significant loss in the salt rejection (98%) of the membranes treated for 1, 2, and 5 min, and the water flux was also found to be statistically similar to its pristine membranes (30 L m^−2^ h^−1^) [29]. Unlike the RO membranes treated at 10 W, water flux and salt rejection declined by more than 50% and 14% after 5 min plasma treatment at 80 W, respectively [29]. Film fractures captured in the SEM images confirmed that performance loss was related to the structural degradation of the TFC treated at 80 W [29]. The TFC membranes salt selectivity dropped below 90%, making it no longer suitable for desalination applications. In the case of UF and MF membranes treated at 25 W, 0.5 mbar, for 2 min, the rise of membrane permeability upon H_2_O plasma treatment was attributed to hydrophilicity enhancements. The water flux increased by 28% for both UF PES and PE from 4856 and 421 L m^−2^ h^−1^, respectively [37], while more than two times for the MF PC and PET which exhibited initial fluxes at 25 and 20 L m^−2^ h^−1^, respectively [32]. However, none of these studies reported the selectivity and antifouling characteristics of the membranes upon the plasma treatment.

#### 2.1.3. Reductive Gas Plasma—Ammonia (NH_3_)

Plasma glows generated from NH_3_ have been previously applied to introduce amine, imine, amide, and nitrile groups onto the surface of membrane materials, rendering a more hydrophilic surface less liable to organic fouling [30,43,44]. Hydrophilic membranes can effectively suppress the hydrophobic interaction between membrane materials, organic solutes, and microorganisms and facilitate the filtration processes [30,45]. Hence, hydrophilization of membrane materials has been one of the focuses of the membrane modification processes [46,47]. Ammonia plasma has commonly been applied individually or in combination with argon/oxygen to generate stable plasma discharge conditions [41]. Past ammonia plasma treatments across different polymeric membranes are summarized in Table 1 (Entries 12–16).

Pure NH_3_ plasma was used to modify laboratory synthesized UF PP [30,45] and PAN membranes [48] (Entries 10–12 in Table 1). The WCA of the PP, modified at 30 W, 0.1 mbar for 4 min, decreased from 128° to 71° [30], and the WCA of the modified PAN decreased from 89° to 13° over the course of 8 min pulse DC plasma treatments with a duty cycle of 70% (450 V and 20 kHz) [48]. Another study used NH_3_/Ar plasma to hydrophilize UF PSf (Amoco Co.), resulting in a reduction of 47% in contact angle analysis comparing to its control (87°) (Entry 15 in Table 1) [41]. Furthermore, NH_3_/O_2_ plasma at a ratio of 3:5 was used to modify UF PES membranes, leading to a complete hydrophilization of the PES surface, whereby WCA was reduced from 66° to nill [43] (Entry 16 in Table 1). The complete hydrophilization was attributed to the polar chemical groups promoted by the incorporation of oxygen gases in the system [28,37,43]. The hydrophilicity enhancement induced by oxygen-associated plasma thus appeared more effective than sole ammonia-associated plasma.

The XPS analysis indicated that the ammonia-associated plasma studies mentioned above modified the chemical structure of the treated membranes by enriching the surface with nitrogen functional groups. In the case of the PSf modified at 60 W, the N/C ratio increased to 0.113 and 0.223 for the NH_3_ and NH_3_/Ar (7:3) plasma-modified PSf, respectively, compared to the pristine PSf contains none nitrogen moieties in its structure (Entry 15 in Table 1) [41]. Furthermore, 22.7% of the C-N functionalities appeared on the PSf upon NH_3_/Ar plasma, which was 13.5% higher than that of the PSf modified by the pure NH_3_ plasma [41]. This study highlighted that the addition of Ar gas facilitated the stability of the NH_3_ plasma discharge and led to a higher degree of nitrogen enrichment onto the PSf membranes.

The impact of NH_3_ and NH_3_/O_2_ plasma on the membrane performance was highlighted using the Entries 12 and 16 from Table 1. The water flux of the UF PP membrane, treated for 1 min with pure NH_3_ plasma at 30 W, increased two-fold in comparison to the pristine PP membranes with an initial flux of 350 L m^−2^ h^−1^ [30]. The flux remained 1.39-fold higher than the PP after exposure to bovine serum albumin (BSA) solution for 24 h at 30 °C (Entry 12 in Table 1) [30]. Furthermore, 100% of flux recovery was achieved for the modified PP after rinsing with water, whereas the pristine PP only recovered 58.2% of its initial flux [30]. In case of the UF PES (Millipore Corporation) treated by NH_3_/O_2_ plasma at 25 W (Entry 16 in Table 1), the water flux of the NH_3_/O_2_ plasma treated PES stayed two times higher than that of the pristine PES control over the course of 180 min filtration experiment [43]. Furthermore, 76% less of protein adsorption was achieved for the NH_3_/O_2_ (3:5) plasma treated PES with a 90 ± 8% flux recovery rate after cleaning with deionized water (DI) water, in comparison with the pristine PES which exhibited 329 ± 94 (µg/cm^2^) protein adsorption and a 63 ± 8% of flux recovery rate only [43]. The water flux and anti-fouling behavior of the pure NH_3_ and NH_3_/O_2_ plasma suggested that the incorporation of nitrogen and nitrogen moieties has increased the permeation performance and protein fouling resistance of the treated membranes [43].

#### 2.1.4. The Mechanistic Overview of the Plasma Gas Processes 

The analysis of the tests shown in this section shed light on the type of plasma source gases and the role of different plasma parameters on the materials properties, which directly relate to the membrane material properties and performance. However, insufficient studies have reported the impact of plasma parameters on the membrane material properties and performance as many as possible. Three of the 16 studies listed in Table 1 have comprehensively investigated the impact of plasma conditions on the material properties of the modified PA RO membranes [7,29]. Material properties such as morphology change, surface wettability and roughness, and surface charge were studied as functions of plasma power and duration. The correlation between these material properties and the performance of the modified ROs regarding permeation and salt rejection was also highlighted in these studies. It is highly recommended that future plasma gas studies aim at revealing the material characteristics as many as possible, especially these are closely related to membrane performance. In general, two main underlying mechanisms affecting membrane properties and performance are (i) chemical incorporation and (ii) plasma etching [49]. 

Ring-opening and chemical substitution are two viable pathways for chemical incorporation as hypothesized in Entries 8 (H_2_O plasma) and 14 (NH_3_ and O_2_/NH_3_ plasma) in Table 1 [32,43]. In case of H_2_O plasma, oxygen, and hydroxyl (OH) radicals can be formed after the high energy electrons generated by plasma bombard of their parent source gases such as O_2_ and H_2_O, respectively. Entry 10 (Table 1) highlighted that oxygen and hydroxyl radicals enhanced the ring-opening of the membrane materials such as MF PC and PET for plasma etching (oxidation) in the presence of H_2_O plasma [32]. Firstly, the ring-opening hypothesis was supported by the XPS spectra as the intensity of the π-π* shakeup satellite band decreased for MF PC and disappeared for MF PET after a 2 min H_2_O plasma treatment [32]. Secondly, Entry 10 (Table 1) monitored the concentration of excited species such as O* and OH* in the H_2_O plasma glow based on the optical emission spectroscopy (OES) analysis [32]. The concentration of O* and OH* excited radicals dropped significantly from approximately 0.7 to 0.2 and 0.9 to 0.4, respectively, when the MF PC was exposed to H_2_O plasma [32]. The formation of carboxylic acid groups as a result of subsequent oxidation of implanted groups was the indication of successful chemical incorporation [32]. OH, and H radicals can also facilitate chemical incorporation via substitution by breaking the C–H and C–C bonds which have low bond energies and replacing the OH and NH_2_ groups to the membrane surfaces in case of H_2_O and NH_3_ plasma, respectively [32,43]. 

Plasma gas etching led to two different morphology changes such as surface roughening and smoothing owing to the plasma parameters such as input power and duration, as well as the material structure of the pristine membrane substrates, based on the studies summarized in Table 1.

Long treatment durations and/or at higher plasma power inputs were found to be the main causes of surface roughening, and membrane pore enlarging, as reported in Entries 4 and 9–11 in Table 1. Prolonged plasma treatments contributed to the secondary reactions between the excited species originated from plasma gas and the radical-sites across the surface of the modified membrane substrates because of plasma activation, which could lead to a rougher surface. Entry 10 reported that the implanted oxidized groups eventually converted to CO_2_ or other volatile species via secondary ring-opening of the functional groups across the surface of the PC and PET membranes as plasma duration increased to 30 min [32].

Surface smoothing effects induced by Ar, He, and H_2_O plasma were reported in cases of modifying PA RO membranes Entries 1–2 and 9 (Table 1), which was likely related to the material structure of the pristine PA RO membranes. During the plasma treatments of these studies, the etching mechanisms were that plasma gas preferentially etched the amine functional groups and subsequently contributing to the secondary cross-linking reactions between the plasma-activated species originated from the membrane surface and plasma-activated membrane surface, leading to a smoother surface [7,40,50].

Nevertheless, plasma gas surface modifications have also faced challenges related to the long-term chemical stability introduced groups after the treatment. Few studies have investigated the long-term chemical stability of such grafting and only seven studies presented in Table 1 investigated this matter (Entries 3, 5–6, 8–10, and 16). Particularly, post plasma oxidation and polymer chains reorientation are two main issues which were shown to cause hydrophobic properties recovery of hydrophilized membranes upon exposure to environmental air, as an unwanted aging characteriztic of plasma treatments [51,52]. 

Post plasma oxidation is a reaction between the unreacted free radicals remaining on the membrane surface and various gas molecules from the air after treatments [51]. Free carbon radicals remained on the modified surfaces are likely oxidized into hydroxyl, carbonyl, carboxyl groups once exposed to the atmosphere. It can be resolved by purging nitrogen into plasma reactors for the 20 s after plasma treatment [7,29], limiting direct reactions between plasma modified membrane surfaces with atmospheric air. 

The second scenario corresponding to the reorientation of polymer chains is likely caused by plasma-generated polar functionalities moving toward bulk polymer [52]. It has been reported that these phenomena tend to be significant and can be detected by the increase of water contact angle after aging. Consequently, the surface regains hydrophobic properties, which is also known as ‘‘hydrophobic recovery’’ [52]. Entries 6–11 and 16 (Table 1) claimed that wetting both sides of membranes could mitigate such issue especially by positioning membranes perpendicularly to the feed flow of gases. 

In summary, plasma gas treatments are an effective technique for wetting the membrane surface and introducing potential chemical functionalities, depending on the types of applied gases and plasma conditions. Substrate degradation caused by etching and the instability of the modified surfaces are two shortcomings adversely affecting the material properties and applications of the modified membranes, which requires plasma conditions testing and long-term monitoring.

### 2.2. Low-Pressure Plasma Polymerization Treatments toward Surface Modification

Plasma polymerization is a process developed for surface modification by producing nanoscale films [14,53], suitable for altering materials surface properties [12,13]. The polymer structure generated by plasma polymerization are cross-linked to generate controlled free-volume [11]. In addition, deposited structures may be pinhole-free and produced from either single or mixtures of monomer precursors allowing for customizable chemistries. In the following sections, the impact of plasma polymerization of amine, carbonyl, allyl alcohol functional groups, and organosilicon—and fluorocarbon—moieties onto polymer membranes will be discussed.

#### 2.2.1. Plasma Polymerization of Amine Monomers onto Membranes

The surface of MF PP [12], UF PSf [12,13], and RO PA TFC [8] membranes have been previously amine-enriched by plasma polymerization. The objectives of these studies were to either produce composite membranes for desalination [13], improve the permeability and fouling resistance of the materials [12], or tune the surface charge of the composite membranes [8,54]. 

Entry 1 in Table 3 aimed at producing selective films for sodium chloride rejection (2000 ppm concentration). The amine-enriched films were deposited onto MF and UF membranes by plasma polymerizing allylamine [13]. Commercial MF PP (Hoechst–Celanese Co.) and laboratory synthesized UF PSf were used as substrates, and the performance of the modified membranes was investigated related to plasma treatment conditions [13]. As seen in Table 2, The water flux of the modified PP decreased by 98.4% compared to the pristine PP. Salt rejection increased from 0% to 92% by fixing plasma power at 10 W and monomer flow rate at 0.8 sccm, only increasing treatment duration from 10 to 60 min [13]. Under the same treatment conditions, flux decreased by 96.7%, and salt rejection rose 95% for PSf ultrafiltration membranes. Furthermore, the pores across the PP surfaces have gradually disappeared while a non-porous surface started forming as plasma duration increase based on SEM images. SEM images also indicated that longer plasma durations induced higher density layers with complete coverage of plasma polymerized layers across the PP surfaces which correlated well with decrease in pure water flux and increase in salt rejection [13]. The performance of both PSf and PP membranes was greatly altered and appeared to feature in the NF and RO range applications.

The performance of the amine-enriched membranes was also studied as a function of plasma power densities and monomer molar flow rates with a focus to reduce polymerization duration. By fixing the molar flow rate at 0.8 sccm and reducing the polymerization duration from 60 to 30 min, water flux of the PP modified at 50 W for 30 min was two times higher than that of the PP modified at 10 W for 60 min, respectively. Salt rejection was maintained at 88% at 50 W, but 4% lower than the membrane modified at 10 W for 60 min [13]. Such an outcome suggested that increasing plasma power can effectively reduce plasma duration. The results suggested that increasing plasma power to 50 W while reducing the treatment time to 30 min can produce dense membranes with similar perforamnce as the one modified at 10 W for 60 min. In the case of PSf membranes, both the water flux and salt rejection slightly increased by 3–4% after changing power from 10 to 50 W for a 30 min treatment [13]. This suggests that increasing plasma power helps reduce the treatment durations and thereby increasing the efficiency of the plasma polymerization processes by cross-comparing the membrane performance of the PSf treated at 10 W after 60 min and the ones treated at 50 W after 30 min [13]. 

Furthermore, the impact of the monomer flow rate on the membrane performance was also investigated. When molar flow rate increased from 0.8 to 1.8 sccm at constant plasma power (10 W) and duration (60 min), the water flux rate of the modified PP increased from 0.1 to 5.5 (L m^−2^ h^−1^), and salt rejection decreased from 92% to 20% (a difference of 72%) [13]. The results indicated that the modified PP was no longer suitable for NF applications after increasing monomer flow rate. A similar trend was observed for PSf membranes. Water flux increased more than four times (from 0.5 L m^−2^ h^−1^) and salt rejection loss by 65% (from 95%) by comparing to the PSf membrane treated at lower monomer flow rate [13]. The underlying mechanism of the significant performance losses in flux and salt rejection at a higher monomer flow rate (1.8 sccm) was attributed to high reactor pressure (40 mTorr) leading to the formation of low homogeneity layers [12,55]. It is expected that the higher the monomer flow rate and reactor pressure, the lower the energy transferred to the monomer molecules, due to the shorter residence time across the plasma reactor [13]. As a result, plasma polymerized films with a low degree of cross-linking were formed in this configuration [12,13]. Similar systematic studies of using plasma polymerization techniques to produce RO composite membranes were extensively performed in 1970–1980s [55,56,57,58], showing great potential in producing RO and NF composite membranes via plasma polymerization route.

The deposition of amine-enriched films was also found to be an effective way to hydrophilize materials in a similar fashion to using reductive gas plasma. The correlation between the material properties and performance of the MF PP (Hoechst–Celanese Co.) membrane was examined as a function of power, duration, and reactor pressure (Entry 2 in Table 3) [12]. The WCA and water flux of the amine-enriched PP membranes decreased by 40% and increased by 50% respectively, in comparison to the pristine PP (108° and 0.8 L m^−2^ min^−1^) upon plasma treatment at 5 W and 5.332 Pa for 10 min [12]. The pore diameter of the modified PP was found to decrease from approximately 240 nm (pristine PP control) to 160 nm. However, its water flux increased by 37.5% compared to the flux of the pristine PP (48 L m^−2^ h^−1^) [12]. The characterization results suggested that the impact of the hydrophilic properties outperformed pore size on the permeability of membrane material, reducing the concern of losing permeability upon the deposition of plasma thin films. Similar to the mechanisms of NH_3_ gas plasma discussed in Section 2.1.3. Reductive gas plasma—ammonia (NH_3_), the membranes became more hydrophilic after the deposition of the amine, imine, amide, and nitrile groups [30,43,44]. The antifouling properties of the amine-enriched PP membranes were also examined in Entry 2 in Table 3. A BSA solution was used as the surrogate organic fouling solution, with an isoelectric point (IEP) at pH 4.5. When BSA filtration was carried out below its IEP (pH = 2), amine-enriched PP rejected 35.6% of BSA. In contrast, BSA rejection of the modified PP raised to 89.8% at pH 7 [12]. The result, therefore, demonstrated that amine-enriched PP membranes have a low fouling potential towards protein under basic conditions. The same phenomena were also observed for PSf UF membranes undergoing plasma polymerization of n-butylamine [59,60] and allylamine plasma [59] by microwave discharge.

**Figure 2:**
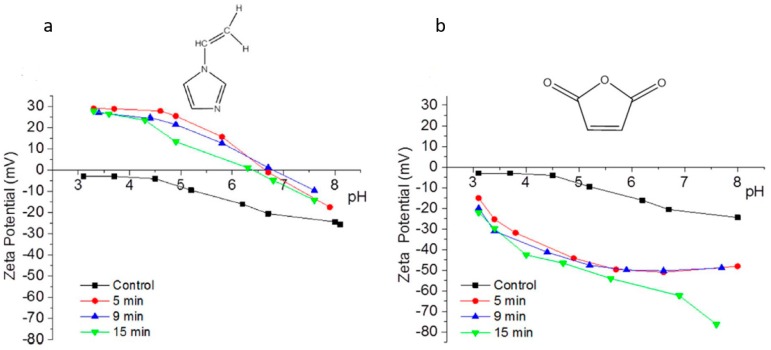
Surface charge analysis of the PA RO TFC membranes deposited with plasma polymerized (**a**) VIM and (**b**) MA. Reproduced from [54], Copyright (2018), with permission from Elsevier.

Entry 3 in Table 3 highlighted the impact of plasma duration on the surface charges of the PA-RO TFC membranes via plasma polymerization of 1-vinylimidazole (VIM) [54]. As opposed to the Entry 2 [12], Entry 3 directly measured the surface charges of the amine-enriched RO membranes by using the streaming potential technique for the first time, instead of speculating surface charges by varying pH of the fouling or the process solutions [12,61]. The surface charges of the modified membranes were reported as a function of plasma duration (5, 9, and 15 min). Figure 2a shows that a sharp increase of the overall surface energy from −25 mV to −5 mV at pH 7, and the IEP of the amine-enriched membranes surfaces dramatically raised to ~pH 7 from ~pH 3 (IEP of the control membrane). The streaming potential results suggested that the deposition of the amine-enriched films has altered the surface properties from a negatively charged surface to a positively charged surface. Figure 2a also demonstrates that the plasma duration may not have a significant impact on the surface properties, since the IEPs of the membrane modified for different durations stayed in a close range of pH 6.3–6.6. Bases on the Entry 3 in Table 3, the material properties of the monomer precursors rather than the plasma duration played a dominant role in the surface charge of the modified membrane [54]. Polymerization of VIM monomers triggered by gamma-ray has shown a similar impact on the PA-RO TFC membranes, rendering largely positive charged (+45 mV) PA-RO TFC membranes which was originally negatively charged (−25 mV) [62].

#### 2.2.2. Plasma Polymerization of Carboxyl Functional Groups onto Polymeric Membranes

Monomers bearing carboxylate functional groups have been utilized for membrane surface modification via alternating current (AC), radio frequency (RF), and microwave powered plasma polymerization [12,35,49,54,63]. Acrylic acid (AAc) has been used to improve the hydrophilicity of MF PP [12] and UF PSf [49] membranes owing to its high volatility, solubility in water and bearing a high ratio of a carboxyl polar group. Alternatively, plasma polymerization of maleic anhydride has been recently applied to customize the surface charge of RO PA TFC membranes [54].

The impact of plasma duration on the material properties of the AAc-enriched films and its relationship with the flux of the modified PP were studied (Entry 4 in Table 3). In this study, material properties and performance were analyszed for the MF PP membranes (Hoechst–Celanese Co.) modified at 5 W and 5.332 Pa (reactor pressure) for different treatment durations (10, 20, and 30 min) [12]. The WCA of the modified PP dropped by 36.1% after 10 min treatment, by 67.6% after 20 min treatment compared to that of the pristine PP 108° [12]. The deposition of the chemical polar groups onto the modified PP was attributed to the increase in hydrophilicity. The FTIR-ATR spectrum showed that a new carbonyl band at 1704 cm^−1^ appeared on the surface of the acrylic acid-treated PP at 5 W and 5.332 Pa for 10 min [12]. The diameter of membrane pores was declined by approximately 50% after the treatment reached 30 min based on SEM images. Furthermore, the hydrophilicity changes were confirmed the water flux tests. The water flux of the modified PP (5 W and 5.332 Pa, 10 min) was 70% higher than that of the pristine PP membrane, supporting contact angle measurements [12]. In accordance to Entry 2 study from Table 3, the results of this study highlighted that the deposition of dense plasma polymerized films, and thus lower membrane porosity, was still be able to increase the water flux of the modified membranes. 

Furthermore, maleic anhydride (MA) was adapted to customize the surface charge of the commercial RO PA TFC (BW 30) as a function of plasma duration (Entry 5 in Table 3) [54]. The streaming potential analysis revealed the surface charge of the modified membranes undergone 5, 9, and 15 min of plasma treatment durations. The streaming potential plot (Figure 2b) indicated that an absolute negative charged membrane was formed upon polymerization of MA. The zeta potential of the membrane declined from −20 mV (control) to approximately −50 mV after 5 min and 9 min treatment, and −80 mV after 15 min treatment at pH 8, suggesting that the negative potential was significantly enhanced due to an increasing amount of the carboxylic acid and thereby the thickness of the films along plasma duration [54]. Correspondingly, the AAc-enriched films also appeared to be negatively charged as it was indirectly approved in the protein fouling tests, as mentioned in the discussion of Entry 2 study from Table 3 [12,64]. 

#### 2.2.3. Plasma Polymerization of Hydroxyl (–OH) Functional Monomers onto Membranes

Plasma polymerization of OH-bearing functional monomers was not as common used as amine (–NH_2_)– and carboxylic acid (–COOH)– bearing ones for membrane modification. One study produced a hydroxyl-enriched films onto the RO PA TFC membranes (SW30HR, DOW Filmtec) upon plasma polymerization of triethylene glycol dimethyl ether (triglyme, C_8_H_18_O_4_), a polyethylene glycol (PEG)-like precursor [65]. Although this precursor does not have any “build in” –OH functionality that can undergo normal radical polymerization, the incorporation of hydroxyl can be relied on the formation of –OH radicals in the plasma-phase.

The impact of plasma duration on the material roughness was highlighted and related it to the RO permeability. The surface roughness of the PA ROs increased from 61.9 nm (PA control) to 66.2, 86.9, and 89.3 nm after 15, 30, and 60 s polymerization, respectively [65]. A total of 44.3% rise in surface roughness after a 60 s plasma polymerization at 1 W RF power, suggesting a roughening effect was induced by the plasma treatment [65]. Such roughening effects were attributed to the chemical structure of the monomer precursor featuring a long chain structure C_8_H_18_O_4_. When the plasma polymerization powered at the relatively low level, such as 1 W, the plasma polymerized coatings prone to feature ether functional group maximally. Thus, the loosely cross-linked, PEG-like units resulted in a rougher surface. The rougher surface can also explain the permeance changes of the plasms-modified RO PAs. The permeance declined 10–15% compared with its control RO PAs, which was measured over the course of a 30 min DI water filtration test using a dead-end filtration system [65]. As a result of the plasma polymerization, the rougher surface with densely cross-linked PEGs reduced the diffusion pathway of PA ROs and led to the permeance reduction [65]. Furthermore, amine– and carboxyl– bearing group plasma polymerization also showed their limitation in increasing the permeability of the RO PA TFC membranes, likely due to the nature of the highly-cross-linked structure of PA surface [8,54].

#### 2.2.4. Plasma Polymerization of Organosilicon and Fluorocarbon Moieties for Membranes Coating

Plasma polymerization of organosilicon and fluorocarbon precursors have been applied to produce hydrophobic films. The purpose of the modification is to decrease the wettability of membranes so that the hydrophobic membranes can be applied to alcohol filtration, pervaporation, gas separation, and absorption processes [66,67,68]. In this section, the deposition of silicon oxides and fluorocarbon thin film onto membranes via plasma polymerization have been briefly reviewed.

##### Deposition of Silicon Oxide (SiO_x_) Thin Films

The correlation between chemical nature of the organosilicon precursors, the material properties, and the gas selectivity of the plasma-modified membrane was investigated by plasma-enhanced chemical vapor deposition (PECVD). FTIR analysis from this study highlighted that the organosilicon monomers with different O/Si atomic ratio could produce organosilicon-enriched films with a different degree of cross-linking [66]. The hexamethyldisiloxane (HMDSO, C_6_H_18_OSi_2_, Si = 2, O/Si = 0.5) precursors with lower O/Si atomic ratio was reported to produce thin films with a partially crosslinked structure. The linear dimethylsiloxane chains (1020–1060 cm^−1^) were found to be partially crosslinked with the Si–CH_2_–Si bridges (1350 cm^−1^), producing the thin films with the most organic silicone-like structure (C_2_H_6_OSi)n [66]. The trimethylmethoxysilane (TMMOS, C_4_H_12_OSi) precursors with a 1:1 O/Si atomic ratio produced more branched, denser, and siloxane-like structured thin films, reflecting by the broader Si–O–Si absorption bands (1060–1080 cm^−1^) [66]. In contrast, the methyltrimethoxysilane (MTMOS, C_4_H_12_O_3_Si) precursors, with higher O/Si atomic ratio (O/Si = 3), led to the formation of the most inorganic silica-like (SiO_2_), highly cross-linked, thin films with low methyl content, and a great quantity of Si–O–Si detected at 1125 cm^−1^ [66]. Hence, the FTIR spectra suggested that organosilicon-enriched plasma films became more cross-linked with less free voids derived from the precursor with a higher O/Si atomic ratio. 

The gas permeation measurements have further supported this theory, the MTMOS-enriched plasma films achieved a He/N_2_ permeance ratio of 15, almost doubled that of the HMDSO derived (7.1) and TMMOS derived (7.8) thin films [66]. The pronounced molecular sieving characteriztics of organosilicon-enriched plasma films derived from and HMDSO and 1,2-bis(triethoxysilyl)ethane have also been reported for gas separation and pervaporation, respectively [67,69,70].

##### Deposition of Fluorocarbon (CF_x_) Thin Films

Fluorocarbon gases or vapors can be utilized to deposit thin films of CF_x_ by plasma polymerization, which have frequently used to decreases the wettability of the membrane surfaces [71]. Fluorocarbon was used to modify perfluorosulfonic acid (PFSA) [72], MF poly(ethylene terephthalate) track-etched membranes (PET-TM) [73,74], and UF PES [75].

The plasma deposition of heptane (C_7_F_16_) film aimed at increasing the hydrophobicity of the commercial perfluorosulfonic acid (PFSA) membrane (GEFC—117 membranes, Golden Energy Fuel Cell Co., Beijing, China) for direct fuel cell application (Entry 9 in Table 3) [72]. The WCA of the modified PFSA membrane increased from 86.9° to 117.3°, completing a transformation from a hydrophilic to a hydrophobic surface, upon a 90 s plasma treatment at 70 W and 0.5 mbar [72]. In contrast, the WCA of the PFSA treated at 30 W and 0.3 mbar for 30 s only increased 6.1% [72]. The XPS results of Entry 9 indicated that such hydrophilicity changes were related to the deposition of (C_7_F_16_)-containing films. The contents of –CCF*x*– in the pristine PFSA (GEFC) have increased from 9.2% to 22.6% following a plasma treatment at 30 W and 0.27 mbar for 30 s by deconvoluting the XPS C1s spectrum. In contrast, the incorporation of –CCF*x*– increased to 32.3% after increasing plasma power, reactor pressure, and treatment duration (70 W, 0.53 mbar, and 90 s) [72]. Such chemical variation was also obtained in another plasma polymerization study using tetrafluoromethane (CF_4_) [75]. The XPS survey and carbon (280–298 eV) spectra showed that fluorine (F) became the dominant component and various fluorocarbon functional groups formed on the modified membrane made of PES, replacing carbon atoms and alkane groups [75].

Entry 9 (Table 3) studied the cross-linking densities of the modified membranes, which has rarely been reported for plasma deposited thin films. The crosslinking degree was calculated based on the deposition rate, the critical thickness and density of the deposited films according to [76]. The degree of crosslinking of the pristine PFSA (GEFC) increased from 28.8% to 73.0% and 76.3% after low level (30 W, 30 s, 0.3 mbar) and high level (70 W, 90 s, 0.5 mbar) of plasma treatments, respectively [72]. The cross-linking analysis suggested that the deposition of denser and highly cross-linked C_7_F_16_-enriched films upon plasma polymerization. 

The methanol permeability of the PFSA treated at 70 W was 0.033 × 10^−6^ cm^2^/s which was 98.0% and 98.6% lower than that of the PFSA treated at 30 W and the pristine PFSA, respectively [72]. Such low methanol permeability achieved at 70 W enable the modified PFSA membranes to be used in the fuel cells at high methanol concentration, which was likely caused by two changes in the chemical structures of membrane materials. Firstly, C_7_F_16_-induced plasma etched sulfonic groups from the pristine PFSA and generated radical site for the subsequent plasma deposition [72]. Secondly, the dense and highly cross-linked C_7_F_16_-enriched films as a result of the deposition of fluorocarbon fragments, thereby effectively hindering the methanol sorption and permeation [72]. 

#### 2.2.5. The Mechanistic Overview of the Plasma Polymerization Processes

Plasma polymerization processes generally involve monomer fragmentation and radical site formation on the membrane surfaces and followed by fragments deposition and polymer formation in the form of thin films [15]. Yasuda and Yasuda proposed that the competitive ablation polymerization was the main mechanism describing the monomer fragmentation in both gas and solid phases [77]. In this primary phase of plasma, as similar to the etching effect induced by the oxidative plasma, the excited species were a mixture of monomer fragments originated from their monomer precursors and sputtered fragments from competitive ablation of the pristine membrane surface and pre-deposited materials, contributing to the formation of radical site for subsequent polymerization and further fragmentation of monomers and excited species [77]. The competitive ablation polymerization was observed in Entries 9 and 12 (Table 3), attributing rougher membrane surfaces mainly controlled by plasma power [72,75]. 

The mechanisms of low-pressure plasma polymerization were reviewed from a chemical point of view by Friedrich [22]. The chemical reactions occurring in the plasma glow have been considered to be complex. Hence, different mechanisms have been proposed with controversy. Three mechanisms, frequently referred in the literature, are atomic polymerization, and radical chain- and ionic chain-growth polymerization [22]. Yasuda proposed the atomic polymerization mechanism to describe the extensive fragmentation of the monomer precursors into excited species with less similarity to the monomer precursors in high-power, continuous wave (CW) powered plasma [53,57]. The chemical reactions between these excited species were expected to take place at the substrate surface, forming highly cross-linked and dense plasma polymer films. Entries 1 and 4 (Table 3) used “Yasuda factor” = W/MF (which W is wattage in J s^−1^; F is the flow rate of monomer in mol s^−1^; M is molecular weight of monomer in kg mol^−1^) [22] to design plasma experiments and to interpret the outcomes.

n(ABCDEF) + plasma (e^−^) → n(A + B + CD + E + F) (Atomic fragmentation) (1)

n(A + B + CD + E + F) → [FCDBAE]n (Recombination polymerization)(2)

Plasma-induced radical chain-growth polymerization involves initiation, chain-growth, and termination processes. Plasma generates high energy electrons mainly to active monomers and produce radicals or radical fragments (fragments●) in the plasma-phase, as well as to generate radicals on the surface of membrane substrates, as an initiation reaction (3). The polymer (P) chain is grown by adding more excited radicals from the plasma phase and terminated by radical recombination [22].

M + plasma (e^−^) → fragment● (Initiation) (3)

fragments● + M → fragment-M●… → fragment-M● + M → Pan● (Chain-growth)(4)

2Pn● → Pan-Pan (Termination)(5)

The following Formulas (6)–(8) describes the plasma-induced ionic chain-growth polymerization similarly to that of radicals [22].

M + plasma (e^−^) → fragment^+^ (Initiation) (6)

fragmentation^+^ + M → Pan^+^ (Chain-growth) (7)

Pan^+^ + M → Pn + 1+ … → Nix-Nix (Termination) (8)

Nevertheless, the homogeneity of the chemical structure has yet been reported to our best knowledge. It is difficult to conclude whether the polymerization induced by plasma is homogeneous or heterogeneous polymerization reactions due to the versatility of the plasma processes. Plasma parameters and the chemical properties of the membrane substrates can all influence the homogeneity of the plasma polymerized films [11]. The homogeneity of the polymerization determines the repetition of the monomer units, which is a crucial factor for governing the membrane material properties and performance. Therefore, the continuity and homogeneity of the microstructure of the deposited thin films need further investigation.

## 3. Atmospheric Pressure Plasma Processes

Research interests in plasma processes have shifted to produce plasma polymers at atmospheric pressure to reduce operational costs associated with vacuum prerequisite [24]. Atmospheric-pressure plasma systems have been developed to render facile operation, continuous, and open system without vacuum prerequisite [23]. In this section, atmospheric pressure plasma processes used for membrane modification and functionalized thin films synthesization were reviewed and are critically discussed. 

### 3.1. Plasma Gas Treatments

The impact of the atmospheric pressure Ar plasma on laboratory synthesized poly(vinylidene fluoride-co-hexafluoropropylene) (PVDF-HFP) microfibrous membranes was studied using a plasma system composed of two rotating plasma jets [78]. The effect was investigated by revealing the WCA of the modified PVDF-HFP as functions of different plasma parameters [78]. The plasma parameters applied as variable or constant in this study are summarized in Table 4. 

As shown in Table 4, the WCA became smaller as plasma duration was prolonged. The WCA of the modified PVDF-HFP drastically dropped from 137° to 30° approximately after 90 s of treatment, and continuously declined to 19° after 150 s of treatment, completing the transition from a hydrophobic into a hydrophilic surface [78]. The hydrophilic enhancement was expected since the formation of polar functional groups, such as hydroxyl and carbonyl groups, were favored upon atmospheric pressure plasma. The formation of these polar functional groups was likely to be the result of the reaction between radicals in the plasma-activated surfaces and oxygen molecules from the ambient environment after the treatment. Such a phenomenon is recognized as oxygen-contamination in the low-pressure processes, whereas it is considered as the oxidation effect induced by the atmospheric-pressure process for membrane hydrophilicity enhancement [23].

The flow rate of Ar gas can also impact the hydrophilicity of the modified PVDF-HFP, reflected by the WCA which reduced to 22° at the highest Ar flow rate (10 slm) used in this study as shown in Table 4 [78]. The underlying mechanisms are attributed to the increased Ar flow rate leading to higher levels of interactions between the activated species and the surface during plasma discharge [78]. Furthermore, in the experiment series 3, the study reported that the hydrophilicity enhancement could not be achieved when the gap between the substrates and the glow was beyond a 10-mm threshold. The WCA remained at 137° similar to that of the pristine PVDF-HFP when the gap was increased to 12.5 mm, which was almost five times higher than that reported for the PVDF-HFP modified at a 10 mm gap [78]. As opposed to low-pressure plasma processes, the activated species could quickly lose their activity due to the higher collision frequency between each other under the atmospheric-pressure conditions [79]. Therefore, the plasma-activated species interacted with each other (and/or air) instead of with the membrane surfaces during the atmospheric-pressure plasmas led to the aggregation of the particles also known as powers formation and inefficient hydrophilicity enhancement [24,79]. A similar impact of the plasma duration and gap between substrates and glow was obtained in the study of modifying the commercial UF PVDF (Millipore Co.) using the mixture of Ar and Methane (CH_4_) in the same plasma set-up [80]. The XPS and FTIR results also supported the hydrophilicity enhancement caused by the formation of oxygen-enriched functional groups. A decline in the element composition (F) of fluorine, from 60.1% to 49.5%, accompanied with an increase in oxygen contents from 0.3% to 6.6% upon atmospheric-pressure plasma [78]. Hence, the methane plasma induced an oxidation effect on the PVDF. In accordance with the modification done on the commercial PVDF, carbonyl (C=O) functional groups appeared in the FTIR spectrum of the modified PVDF, which were previously undetected on the surface of the pristine PVDF [80]. The membrane performance of the modified PVDF was also altered after atmospheric-pressure plasma treatments. The hydrophilicity enhancement induced by atmospheric-pressure plasma was further confirmed by the pure water flux measurements. A 60% flux enhancement of the modified PVDF was obtained at transmembrane pressure 1.4 bar [80]. 

### 3.2. Plasma Polymerization Modification by Thin Film Deposition at Atmospheric Pressure

The choice of chemical monomer precursors used in the atmospheric-pressure plasma polymerization has been limited to the evaporation rate of the applied monomer, which consequently affects the deposition rate of the activated chemical fragments upon plasma discharge. Using atmospheric-pressure plasma remains largely focused on modifying the hydrophilicity of membrane surfaces. For such purpose, carboxyl and organosilicon monomers have predominately been utilized [67,81,82,83,84]. Since vacuum cannot be the driving force, inert carrier gases such as Ar and He have been commonly used to deliver monomer precursors into the plasma chamber through a by-pass monomer chamber. Hence, the flow rate of the carrier gases became a new parameter attributing to the deposition rate, and material properties of the product plasma polymer films, which needs to pay attention to during the atmospheric-pressure processes.

#### 3.2.1. Atmospheric Plasma Polymerization (APP)

##### Deposition of Carboxyl-Enriched Films

Monomers bearing carboxylic acid (–COOH) functional groups such as acrylic acid and maleic anhydride, which have frequently been used in the low-pressure plasma processes, were predominately applied alone or in combination.

Acrylic acid has been successfully polymerized by using plasma jet or dielectric-barrier discharge within in a system where Ar or He was applied as carrier gases [63,81,82]. One of the acrylic acid polymerization studies investigated the influence of the carrier gas type (Ar or He) and plasma duration (1–20 min) on the chemical properties, morphology, and battery performance of the PP membrane as a separator in the lithium-ion battery (LIB) [81]. Input voltage, the flow rate of carrier gas and oxygen were fixed at 30 kV, 0.7 m^3^ h^−1^, and 0.1 m^3^ h^−1^, respectively [81]. 

The WCA of the commercial PP membranes decreased from 112° to 63° upon a 20-min acrylic acid plasma used 0.7 m^3^ h^−1^ Ar and 0.1 m^3^ h^−1^ O_2_ as carrier gas (labeled as Ar/O_2_/AA plasma). Under the same plasma treatment conditions, the WCA of the modified PP dropped to 39° when the carrier gas was switched to 0.7 m^3^ h^−1^ He and 0.1 m^3^ h^−1^ O_2_ (labeled as He/O_2_/AA plasma). The WCA results implied that hydrophilicity enhancement was more effectively carried out under He and O_2_ environment, likely due to the deposition of a higher degree of oxygen-rich functional groups (such as OH and C=O). Such speculation was indicatively and statistically supported by FTIR and XPS analysis, respectively. The ATR-FTIR spectra of the PP modified upon a 10-min He/O_2_/AA plasma showed a much stronger absorbance band at 3500–3000 cm^−1^ and 1700 cm^−1^ corresponding to the –OH group in the –COOH and the carbonyl group (C=O), respectively, compared to that of the samples upon a 10-min Ar/O_2_/AA plasma. Furthermore, the XPS analysis revealed that He/O_2_/AA plasma led to an atomic ratio of 0.46, four times higher than the pristine PP. In contrast, the O/C ratio achieved upon Ar/O_2_/AA plasma was only three times higher than the pristine PP [81]. The best fitting curve of the C1s peaks also demonstrated that He/O_2_/AA plasma enhanced not only the quantity of the oxygen-enrich functional groups but also attributed to the formation of new functional groups such as C=O at 286.2 eV, which has not been detected in the spectra of the pristine PP and Ar/O_2_/AA modified membranes [81]. Although introducing O_2_ can enhance the incorporation of oxygen-rich functional groups across the modified substrates, O_2_, which was also considered as impurities, influences the density of metastable species that govern the stability of plasma discharge under atmospheric pressure [85]. Moreover, the atmospheric-plasma plasma treatments using He as a carrier gas were anticipated to present more homogeneity than the ones using Ar, owing to the high energy and long lifetime of the He metastables in the discharge region [24]. For example, the He 2^3^S state has a potential energy of 19.82 eV and a lifetime of 7900 s [86], whereas Ar ^3^P_2_ state has a potential energy of 11.5 eV and a shorter lifetime than He [24,87]. Hence, less He metastables were anticipated to be quenched by O_2_ and most of He metastables were able to transfer their energy to the monomer and the surface of the modified PP by collision, which explained the formation of C=O groups on the surface of the modified PP membranes upon He/O_2_/AA plasma, but not in Ar/O_2_/AA plasma. 

The morphology change of the PP membrane was studied as a function of Ar/O_2_/AA and He/O_2_/AA plasma duration (1 to 20 min). The SEM images showed that nanofibers connecting PP islands were gradually cleaved as the Ar/O_2_/AA plasma duration increased up to 20 min, and thereby the porosity of the PP membrane enlarged along the treatment [81]. In contrast, the porous structure of the PP undergone He/O_2_/AA plasma was gradually disappeared and replaced by the nonporous film as plasma duration increased [81]. The pore size measurements were used to statistically back up the observation from SEM images. The average pore size of the PP decreased from 57.8 (control) to 27.5 nm after 10 min Ar/O_2_/AA plasma, but then sharply increased to 180 nm after 20 min treatments [81]. In contrast, a negative linear correlation between the plasma duration and pore size was obtained for the PP upon a 20-min He/O_2_/AA plasma. The average pore size dropped from 57.8 (control) to 10.0 nm [81]. Furthermore, the average roughness of the PP decreased from 68.91 to 52.73 nm and 46.16 nm after 10 min Ar/O_2_/AA and He/O_2_/AA plasma, respectively [81]. The morphology analysis together implied that Ar gas had enhanced the etching mechanism, whereas He carrier gas facilitates the growth of the acrylic acid deposition during plasma treatments [24]. The relatively stronger etching impact induced by Ar plasma was likely caused by O_2_ quenching, leading to a less stable plasma discharge compared to He plasma treatments [85].

The battery performance of the LIB assembled with pristine and modified PP membranes was investigated by the coulombic efficiency and discharge capacity. The coulombic efficiency represents the ratio of the discharge to charge capacity of the LIB cells for a series of recharging cycles. The coulombic efficiency of the LIB assembled with the modified PPs by Ar/O_2_/AA, and He/O_2_/AA plasma maintained at about 99.0% and 99.5%, respectively, compared to the pristine PP (98.5%) [81]. Furthermore, the discharge capacity of the LIB assembled with the modified PPs by both plasmas was stabilized at a level above 120 mA h g^−1^ after 50 recharging cycles. In contrast, the discharge capacity of the LIB assembled with the pristine PPs was 120 mA h g^−1^ initially, then gradually decreased to 100 mA h g^−1^ after 50 cycles. Based on the battery performance, the deposition of acrylic acid films alleviated the electronic impedance interface and facilitated the ionic conductivity, which could be attributed to the effective electrolyte retention as a result of the 10-min modified PPs with a reducing pore size [81,88,89]. 

Maleic anhydride has been commonly used to produce the carboxylic acid-enriched film in the atmospheric-pressure processes. One of the atmospheric-pressure plasma studies copolymerized maleic anhydride (MA) and acetylene (C_2_H_2_) for biomaterial application [83]. The hypothesis was that the stability of the carboxyl-enriched film could be enhanced by incorporating unsaturated hydrocarbons [83]. Hence, the degree of cross-linking and carboxyl retention was investigated as a function of the MA:C_2_H_2_ ratio (0.020, 0.037, 0.055, and 0.110), by adjusting the flow rate of the MA vapor and C_2_H_2_ gas [83].

The water stability of the carboxyl-enriched films prepared from different MA:C_2_H_2_ ratios was studied by monitoring the thickness changes of the films after exposing to water for different durations (1, 24, and 128 h). The thickness of the films deposited at the lowest MA:C_2_H_2_ ratio (0.020) increased by 20% to 687 nm. SEM images showed no morphological changes to the deposited films after immersion in water. The carboxyl-enriched films produced from low-pressure plasma polymerized MA was reported to have similar swelling behaviors after exposing to water, as the highly cross-linked structure prevented the dissolution of the film yet the hydrophilic surface still attracted water molecules [90,91]. Although the plasma polymerized films deposited at MA:C_2_H_2_ = 0.037 exhibited a rather constant thickness of ~544 nm after exposure to water for different durations, SEM images showed that sub-micrometer pores started to appear across the surface due to aging [83]. Furthermore, as the MA:C_2_H_2_ ratio increased further, the thickness of the deposited continued to reduce significantly. The films deposited at the highest MA:C_2_H_2_ ratio lost 70% of its thickness after 1 h dissolve within 128 h immersion [83]. At the same time, agglomerated nanostructures appeared with sub-micrometer porosity as revealed across the SEM images [83]. The deposited films prepared from the MA:C_2_H_2_ mixture with a ratio higher than 0.037 featured low cross-linking structures and incorporated non-polymerized MA monomers which were two reasons for the high solubility and instability of the polymers. 

At low MA:C_2_H_2_ ratio of 0.020, the FTIR spectra of the deposited films exhibited a higher intensity of carboxylic acid (–COOH) peak compared to the anhydride peak (C–O–C), whereas the carboxyl peak at ~1730 cm^−1^ was almost negligible compared to the anhydride band when the MA:C_2_H_2_ ratio increased to 0.110. Hence, the FTIR spectra suggested that the degree of fragmentation of MA was low at high MA:C_2_H_2_ ratio. Furthermore, the presence of bands corresponding to OH, sp^2^ C–H, and sp^3^ C–H between 2800–3750 cm^−1^ implied that the incompleted fragmentation and non-polymerization of the copolymerization at high MA:C_2_H_2_ ratio, speculating that the deposited films consisted of polymerized MA precursors or oligomers. Such a hypothesis was further justified by the AFM analysis used to reveal the topography of the carboxyl-enriched films prepared at high MA:C_2_H_2_ ratio. For a 10 min plasma deposition, not only the thickness of the films increased from 687 to 938 nm, the rms of height differences (σ) and autocorrelation length *T* of the surface roughness also increased from 70 to 133 nm, and 65 to 113 nm, respectively [83]. The rise of the surface roughness supported the idea that the deposition of carboxyl films included a large proportion of protrusions such as non-polymerized MA or oligomers as partially polymerized MA.

##### Deposition of Silicon Oxide (SiO_x_) Thin Films

Analogously to the low-pressure plasma studies, organosilicon precursors were also applied in atmospheric-pressure plasma processes to produce gas permselective films on top of a microporous silica composited membrane. The silica composited membranes were prepared by casting a silicon dioxide-zirconium dioxide (SiO_2_-ZrO_2_) intermediate layer via a sol-gel method on the surface of a porous α-alumina capillary tube (NOK, Co.) [67]. Microporous organosilicon-enriched films were produced by atmospheric-pressure plasma enhanced chemical vapor deposition (AP-PECVD) using HMDSO as the precursor [67].

The chemical composition of the plasma polymerized HMDSO films was analyzed by XPS and FTIR. By comparing the elemental ratio of C/Si and O/Si listed in Table 5, the films featured silica-like structures with a O/Si ratio of 1.98 and a low level of carbon (C/Si = 0.11) using pure Ar as carrier gas [67]. The O/Si ratio was slightly higher for the films deposited using the O_2_/Ar gas mixture as an anticipated outcome of the interaction between the ground-state O atoms, metastable O_2_ molecules, ozone, and HMDSO precursors [67]. On the one hand, oxygen-bearing chemical groups such as Si-OH and OH were formed across the films deposited using an O_2_/Ar gas carrier mixture [67]. On the other hand, the films deposited using N_2_/Ar has the C/Si ratio 0.58–0.59 higher than that using pure Ar and O_2_/Ar gas, and the O/Si ratio (1.57) most differentiated from SiO_2_ (2) [67]. Furthermore, a variety of carbon vacancies corresponded to N–H, C–H, C≡N, N–H, Si–C, and Si–O also appeared across the FTIR spectrum, suggesting that a higher degree of HMDSO polymerization was taken places using N_2_/Ar as working gas compared to the other two during the atmospheric plasma treatments.

The morphology of these organosilicon-enriched films varied in accordance to their chemical structure. The SEM images indicated that the films deposited in the pure Ar and O_2_/Ar gas presented some similar features such as a different degree of discontinuity and the embedment of granular shaped particles. Particularly, film fractures were observed from the SEM images of the films grew in the O_2_/Ar gas, likely due to the O_2_ plasma etching effect as discussed in the low-pressure oxygen plasma [49]. 

**Figure 3:**
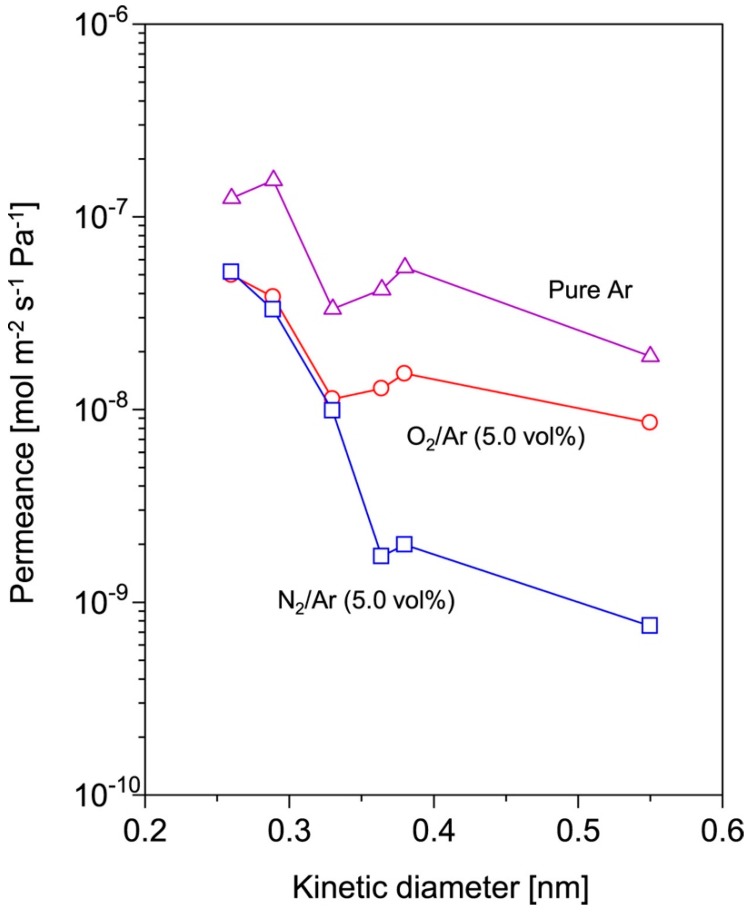
Single gas permeances of the composited membranes prepared by AP-PECVD with different working gases as a function of the kinetic diameter of the permeating molecules. Reprinted from [67], Copyright (2018), with permission from Elsevier.

Furthermore, the composite membranes composed of organosilicon-enriched films, SiO_2_-ZrO_2_ intermediate layer, and alumina as a bottom substrate, were synthesized for gas separation application. The impact of different plasma carrier gases (Ar, O_2_/Ar, and N_2_/Ar) on the gas permselectivity of the organosilicon-enriched films was studied against the kinetic diameter of the He, H_2_, CO_2_, N_2_, CH_4_, and SF_6_ gases. The gas permeance of the films prepared from N_2_/Ar was much higher than that using the O_2_/Ar and pure Ar gases. The He permeance of the films prepared from N_2_/Ar was 0.52 × 10^−7^ mol m^−2^ s^−1^ Pa^−1^, which is lower than that 1.52 × 10^−7^ mol m^−2^ s^−1^ Pa^−1^, the He permeance achieved by the films prepared from O_2_/Ar. Furthermore, the gas permeance decreased with increasing kinetic diameter of the gases, indicating the molecular sieving properties of the films prepared from N_2_/Ar gas, as indicated in Figure 3 [67]. The films prepared from N_2_/Ar also indicated higher gas permselectivity against different permeating gas molecules. The films prepared from N_2_/Ar provided highest permeance ratio of He/H_2_ as 1.6, indicating more He over the H_2_ molecules passed through the membranes [67]. In contrast, the permeance ratio of He/H_2_ was 1.3 and below 1 for the films prepared from O_2_/Ar and pure Ar, respectively, and the permeance ratio of the rest gases varied as less significant compared to the films prepared from N_2_/Ar working gas [67]. The poor gas permeance and permselectivity of the films prepared from O_2_/Ar and pure Ar were likely due to the film fractures and discontinuous film structure as revealed by the SEM images.

#### 3.2.2. Aerosol-Assisted-Atmospheric Plasma Polymerization (AA-APP)

Aerosol-assisted atmospheric plasma polymerization (AA-APP) processes have been developed with the intention of optimizing the deposition efficiency. The plasma polymer generated from atmospheric pressure faces few challenges. The formation of powder products rather than the homogenous thin films is a critical challenge due to the high-frequency collision between activated species in the gas phase [23,24]. Atomic nucleation consequently takes places in the gas phase rather than on the surface of the substrates [24]. Another challenge is related to the limited amount of applicable organic monomer precursors. Monomers such as acrylic acid and HMDSO with high vapor pressure have still been predominately used in the atmospheric-pressure plasma processes owing to their volatility [67,81,82,83]. Limited choice of chemical precursors has hindered the versatility of the atmospheric-pressure plasma processes [23]. Hence, the invention of the AA-APP processes can effectively overcome these two challenges. In the AA-APP system, a pneumatic or ultrasonic atomizer is added to nebulize the monomer precursors into droplets and to deliver them into the DBD plasma discharge zone. The dispersion of the monomer precursors in the form of droplets contributes to the formation of homogeneous films due to radical–radical recombination between the plasma activated precursors and substrate surface [92,93]. Furthermore, with the aid of the atomizer, the chemical precursors with low-volatility and high molecular weight, and a mixture of organic monomers and inorganic nanoparticles can be easily nebulized and dispersed in the atmospheric-plasma system [93,94,95,96]. The deposition of the poly(ethylene glycol) (PEO)-enriched films produced from AA-APP process was symmetrically investigated by studying the effect of the applied voltage and the flow rate of aerosol/carrier flow on the chemical composition of coatings deposited [95]. When frequency and total flow fixed at 27 kHz and 9 slm (He carrier gas flow rate at 5.85 slm, and aerosol flow rate at 3.15 slm), the applied voltage varied at 6.5–8.5 kV, equivalently to 8–13 W power, the deposition rate consequently increased from 18 ± 3 to 38 ± 2 nm min^−1^ [95]. The XPS results showed that C–C content and C/O ratio, increase with increasing the applied voltage. However, C1s fitting data showed that a slight reduction of the PEO-featured peak at 286.2 eV dropped slightly from 65 to 60% as the applied voltage increased from 6.5 to 8.5 kV [95]. The C1s fitting data implied that the fragmentation of PEO precursors was relatively high at 8.5 kV and resulted in highly cross-linked PEO films. The influence of the aerosol/carrier flowrate was studied in a range of 8 to 10 slm, with the rest of parameters were fixed at 27 kHz, 8.5 kV, and the aerosol flow at 3.15 slm for 5 min deposition [95]. By increasing the aerosol/carrier flow rate from 8 to 10 slm, the PEO-featured peak in the XPS C1s fitting spectra decreased from 70 to 54% [95]. The loss of the PEO character was caused by the reduced amount and quicker passage (shorter residence time in the plasma zone) of the aerosols as consequences of increasing the aerosol/carrier flowrate [95]. 

Another focus of the AA-APP processes has been towards the synthesis of organic–inorganic nanocomposite (NC) coatings. The NC coatings made of zinc oxide (ZnO) nanoparticles (NP) and n-octane were prepared by a sinusoidal AC powered, high voltage (26 kHz) plasma [93]. The thin film characterization showed that the compatible incorporation of polyethylene (PE)-enhanced organic coatings and oleate-capped ZnO NPs. The oleate was used to prevent the NPs aggregation in the suspension mixture before the plasma polymerization. Firstly, the ZnO character band at 420 cm^−1^ appeared, and the absorbance intense of the PE character bands representing sp^3^ C–H, CH_2_, and CH_3_ groups were enhanced in the FTIR spectra of the NC coatings samples (formed upon a 10 min DBD AA-APP of a 3% oleate-capped ZnO NPs dispersion in n-octane), by comparing with that of the n-octane films and oleate-capped ZnO NPs upon AA-APP treatments [93]. Furthermore, the XPS C1s and O1s spectra showed that the hydrocarbon components (C–C/C–H at 285 ± 0.2 eV, ca. 94%) and oxygen in ZnO structure (530.2 ± 0.2 eV, ca. 66%) were the two dominant components in the NC coatings [93]. The transmission scanning electron microscopy (TSEM) further demonstrated that ZnO NPs agglomerates were covered by approximately 10–20 nm organic layers under high-magnification dark-field, suggesting that the NC coatings consisted of both organic PE-like layers and inorganic ZnO NPs features [93]. Investigation on using such NC coatings for photocatalytic, self-cleaning, and antistaining applications is under the way [93]. The AA-APP method was also used for producing NC coatings composed of HMDSO and aluminum–cerium oxide (AlCeO_3_) NPs for corrosion protection [97]. 

#### 3.2.3. The Mechanistic Overview of the Plasma Gas and Polymerization Processes at Atmospheric Pressure

The basic mechanisms of the atmospheric pressure plasma treatments including both gas and polymerization are very similar to that of the low-pressure plasma reatments. Especially, both plasma jet (Entries 1, 2, and 4) and DBD (Entries 3, 5, and 6) dicharge systems applied by the studies summarized in Table 6 are plasma glow discharge systems which can be explained by ionziation wave mechanism [24,98,99,100]. However, new mecahisms have to be highlighted when parameters such as plasma carrier gases (He or Ar) and monomer in areas ols form were involved simultaneously. Especially, a major difference of the atmospheric pressure plasma polymerization processes was the radical sources at plasma initiation stage. High energy electrons generated by plasma mainly react with monomer precurors in the plasma-phase, subsequently induced either the radical—or the ionic—chain polymerization at the surface of the modified membrane.

M + plasma (e^−^) → fragment●/fragment^+^ + A → fragment-A + A^+^ + e^−^ (9)

In addition to these mechanisms, the atmospheric pressure conditions add a significant degree of instability due to the quenching phenomenon as a result of high degree of collisions between plasma activated species. Unlike low-pressure plasma treatments, not only atomic ions but also mostly ionic clusters play a role during the plasma treatments carried out at atmospheric pressure [100,101]. Such ionic clusters can be characterized by mass spectrometry diagnostics [100,101]. It should be highlighted that mass spectrometry is a better diagnostic for revealing the active atomic ions and ionic clusters generated by atmospheric-pressure plasma treatments than optical emission spectroscopy (OES) [100,101]. OES analysis is a valuable technique to reveal the excited states of radicals and species based on the ground states of the precursors for the low-pressure plasma treatments [102]. In contrast, such analysis becomes questionable since quenching processes play a major role in the atmospheric-pressure plasma treatments. Among many possible routes, the interaction between the excited species and high-energy metastable atoms (A) originated from the plasma carrier gases and atmospheric environment has been accepted.

M + plasma (e^−^) → fragment●/fragment^+^ + A → fragment-A + A^+^ + e^−^ (10)

However, the formation of dense and homogeously uniformed polymer films was limited by the low mean free path of the excited species in the plasma glow at atmospheric pressure [23,24]. The low mean free path lead to an increasing number of collisions among excited materials, resulting in an over-heating of plasma treatments which may burn the membrane substrates [24]. Moreover, when the collision frequency of the excited species became high, these excited species would be coalesced in the gas phase rather than at the interface of gas and membrane surfaces, forming dissatisfied non-dense and powder-featured plasma polymers [23]. The aerosol-assisted-atmospheric plasma polymerization (AA-APP) was developed to ease the drawbacks derived from low mean free path. With the aid of atomizers, most of the nebulized monomers would have a high chance to be delivered near the substrate surfaces. Hence, the plasma polymerization could take place at the interface of gas and substrate as wanted under atmospheric conditions [23]. Recent AA-APP studies have been focused on the introductory method of the monomer aerosols to improve the density and homogeneity of coatings and thereafter the deposition rate for large scale industry application [23].

## 4. Conclusions and Prospects

Plasma gas and polymerization processes under low-and atmospheric-pressures were critically reviewed, with an emphasis on the impact of plasma mechanisms on both membrane material properties and performance. Examples of membrane modification using different plasma gas sources and monomer precursors at low- and atmospheric-pressures were described, respectively. Material properties such as WCA, chemical composition, morphology, roughness, and surface charge controlled by plasma power, duration, and the flow rate of plasma gas, monomer precursors, and carrier gases were elucidated. The water flux and selectivity performance of the modified membranes were discussed in relation to the changes of material properties. 

Chemical reaction occurring in low-pressure plasma gas treatments included ring-opening and chemical substitution induced by plasma etching/oxidation and followed by chain scission, further oxidation, hydrolysis, and alcoholysis to complete the chemical implantation. The chemical implantation was also under the influence of the excited species originating from surrounding environment for the plasma gas modification carried out at atmospheric pressure. The mechanisms of plasma polymerization were much more complex which involved plasma initiation, chain-growth, and polymer formation (termination of chain-growth). The mechanism of plasma initiation was very similar to that of plasma gas treatments. With the involvement of monomer precursors, plasma polymer started to develop via either plasma-induced atomic or ionic polymerization and terminated by radical recombination, similar to conventional radical polymerization. Similarly, to plasma gas treatments, plasma polymerization at atmospheric pressure also has to consider the metastable atoms and ions originated from carrier gases and the ambient environment. At both low- and atmospheric pressure, plasma gas treatments have shown great potential in enhancing the membrane surface wettability and permeation, excepting He gas—due to its low molecular weight and ionization energy. The impacts of plasma polymerization on membrane performance were more complicated than plasma gas which were not only controlled by plasma parameters but also influenced by the material properties of the membrane substrates. The application of the APP and AA-APP modified membranes has limited to gas separation and battery, studies on liquid separation of the APP or AA-APP modified membranes are anticipated in upcoming years. Furthermore, despite the plasma modification approach can alter membrane surface, structure, and performance simultaneously, the stability, reliability, and reproducibility of the products require further investigation and long-term surveillance. Atmospheric pressure plasma treatments still have high scope for application as large-scale membrane modification processes, since such systems have been successfully applied in various industries such as automotive and textiles manufacturing, for metal corrosion protection, as surface cleaning, towards adhesive stability enhancement, and as thin films coatings [103,104].

## Figures and Tables

**Table 1:** Material properties and performance of the polymer membranes upon low-pressure plasma gas treatments (continued on the following three pages).

Entry	Plasma Treatment	Plasma Conditions	Membrane	Flux (L m^−2^ h^−1^)	Salt Rejection (%)	Water Contact Angle(°)	Surface Charge (pH)	Roughness RMS (nm)	Ref.
1	Ar	10, 50, or 80 W RF power; 0.2 mbar; 1, 5, 15, or 30 min	RO PA Hydrophilic BW30 TFC (Dow Filmtec Corp.)	Raised by 22% (10–50 W) and then dropped by 76% (80 W; 30 min) compared to control 45	98 (control) to 97 (10–50 W; 1–15min), ~60% (50 W; 30 min), ~6% (80 W; 30 min) ^1^	Declined ~15 with increasing power density and time from 60	Negative charge from pH 3 to 8 for both control and modified	Declined to ~40 (80 W; 30 min) from 60 (control)	[7]
2	He	10 or 80 W RF power; 0.2 mbar; 1, 2 or 5 min	RO PA Hydrophilic BW30 TFC (Dow Filmtec Corp.)	Raised by 66% (10 W, 5 min); by 25% (80 W, 5 min) compared to pristine RO PA 30	Maintained at 98% ^1^	47 (PA control) to 10 (5 min 10 W)	NR	63 (PA control) to 58 (10W, 2 min); to ~40 (80 W, 5 min)	[29]
3	O_2_	30 W RF power; 10 cm^3^/min O_2_ vapor flow rate; 0.1 mbar; 0–10 min.	UF PP Hydrophobic Laboratory synthesized	Increased 30% after 1 min, and 15% after 4 min, compared to its control 350	NR ^2^	128 (control) to 72 after 9 min treatment	NR	NR	[25]
4	O_2_	25 W RF power; 0.1 mbar; 1–5 min.	MF PP Hydrophobic Osmonics, Germany	Increased >50% after 5 min, compared to its control 243	NR	135 to 20 after 5 min treatment	NR	NR	[26]
5	O_2_ or Ar	100 W; 20 kHz frequency; 0.13 mbar; 0–6 min.	RO PA Hydrophilic Laboratory synthesized	Increased more than 2.5 times its control (20) after 3 min O_2_ plasma; whilst only 4% higher than its control after 3 min Ar plasma	NR	77 (Control, laboratory synthesized) to 70 after 2 min, and to 44 after 6 min O_2_ plasma; to 69 after 6 min Ar plasma	NR	NR	[40]
6	CO_2_	5, 10, and 20 W RF power; 0.2 mbar; 10–300 s	UF PSf Hydrophobic US Filter, Inc.	Increased 2.3-fold compared to control (175) modified at 10 W	NR	94 (control) declined to 47 (10 s), to 15 (30 s), and to 0 (60 s and 180 s) at 10 W ^3^	NR	NR	[27]
7	CO_2_	20 and 35 W RF power; 0.2 mbar; 0.5–15 min	UF PES Hydrophobic Millipore Corporation	NR	NR	66 (control) to 0, with the water drop, disappears within 25 s (35 W, 30 s) and 75 s (20 W, 30 s)	NR	NR	[28]
8	H_2_O	25 W RF power; 0.5 mbar; 2 min	UF PSf Hydrophobic US Filter, Inc.	NR	NR	86 (control) to 0	NR	NR	[42]
9	H_2_O	25 W RF power; 0.5 mbar; 2–4 min	UF PES and PE Hydrophobic Millipore Corporation	Increased 28.3% for PES (compared to its control 4856) and 28.4% for PE (compared to its control 421)	NR	63 (control) to 0 for PES, 123 to 0 for PE ^3^	NR	NR	[37]
10	H_2_O	25 W RF power; 0.7 mbar; 2 min	MF PC and PET Hydrophobic Sterlitech Corporation	Increased from 25 (control) to 68 for PC, and raised from 20 to 45 for PET	NR	97 (control) to 38 for PC, 59 (control) to 27 for PET	NR	NR	[32]
11	H_2_O	10 and 80 W RF power; 0.2 mbar; 1, 2, and 5 min	RO PA Hydrophilic BW30 TFC Dow Filmtec Corporation	Declined by >50% compared to pristine RO PA 30	98–84% (80W) ^1^	Declined to ~11 (modified –10 W) ~20 (modified –80 W) from 47 (control)	Negative charged from pH 3 to 8 for both control and modified	Declined to 58 (10 W), ~36 (80 W) from 63 (control) after 2 min	[29]
12	NH_3_	30 W RF power; 0.1 mbar; 0–8 min.	UF PP Hydrophobic Laboratory synthesized	Two times higher than control (350) for 1 min-treated sample, 20% higher 8 min treated samples	NR	128 (control) to 54 after 8 min	NR	NR	[30]
13	NH_3_	30 W RF power; 0.1 mbar; 4 min.	UF PP Hydrophobic Laboratory synthesized	NR	NR	128 (control) to 71 under 10 Pa; to 90 under 104 Pa	NR	NR	[45]
14	NH_3_	450 V Pulsed DC power supply; 20 kHz; 0.12 mbar; 9.6 cm^3^/min; three duty cycles (Dt), 30%, 50%, and 70%; 0–8 min	UF PAN Hydrophobic Laboratory synthesized	32% higher than PAN (control ca. 55) after 1 h oil-water filtration test ^4^	NR	89 (control) to 29 (8 min, 30% Dt), to 13 (8 min, 70% Dt)	NR	NR	[48]
15	NH_3_, NH_3_/Ar	60 W microwave power; 125 Hz frequency and 25% of duty cycle; 1 mbar; 10 cm^3^/min Ar flow rate; 0–10 min.	UF PSf Hydrophobic (Amoco, CO., US)	NR	NR	87 (control) to 46 (not specified in the study)	NR	NR	[41]
16	NH_3_, NH_3_/O_2_	15–120 (25) W RF power; 0.07–0.53 mbar; 2–25 (3) min	UF PES Hydrophobic Millipore Inc.	70% (25 W, 3 min, 3:5 NH_3_/O_2_) higher than PES (control ca. 260) after 30 min PW filtration	NR	66 (control) to 0 (25 W, 3 min, 3:5 NH_3_/O_2_)	NR	NR	[43]

^1^ 2 h 2000 ppm NaCl solution under 15 bar, 25 °C; ^2^ NR: not report; ^3^ Water drop applied to the surface disappeared within 2 s; ^4^ Permeate flux for 500 mg/Loil-water emulsions under 276 kPa.

**Table 2:** Membrane performance of the amine-enriched PP and PSf membrane under different plasma polymerization conditions (numerical data are extracted from [13]).

Constant Conditions	Variable Conditions	PP Membranes		PSf Membranes	
		Water Flux (L m^−2^ h^−1^)	Salt Rejection (%)	Water Flux (L m^−2^ h^−1^)	Salt Rejection (%)
10 W/0.8 sccm	pristine	15.5	0	15.2	0
	After 60 min	0.1	92	0.5	95
0.8 sccm/30 min	10 W	~0.7	~88	~1.5	85
	50 W	0.2	~88	~1.55	88
10 W/60 min	0.8 sccm	0.1	92	0.5	95
	1.8 sccm	5.5	20	~2.75	30

**Table 3:** Material properties and performance of the polymer membranes upon low-pressure plasma polymerizations (continued on the following three pages).

Entry	Plasma Polymerization Treatment	Plasma Conditions	Application	Flux (L m^−2^ h^−1^) ^d^	Salt/Solute Rejection (%)	Water Contact Angle(°)	Surface Charge - IEP (pH)	Roughness RMS (nm)	Ref.
1	Allylamine 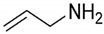	10–50 W RF power; monomer flow rate = 0.6–1.8 sccm (standard cm^3^/min); 1–60 min;	MF PP (Hoechst-Celanese Co.) and UF PSf Laboratory synthesized Hydrophobic	Declined 91% for PP, and 96% for PSf (10 W, 0.8 sccm, 50 min), compared to its control 15.5	Salt rejection of PP and PSf increased 90% and 86% from 0%, respectively ^1^	NR ^2^	NR	NR	[13]
2	Allylamine	10–50 W RF power; reactor pressure at 0.053, 0.093 and 0.133 mbar; 10–30 min	MF PP Hydrophobic (Hoechst-Celanese Co.)	Increased by ~38.5% (5 W, 5.332 Pa, and 10 min), compared to its control 48	89.8% of BSA rejection at pH 7 (5 W, 5.332 Pa, and 10 min) ^3^	108 (PP control) declined 38 (5 W, 5.332 Pa, and 30 min)	Negative charged at pH 7	NR	[12]
3	1-vinyl(imidazole) with Ar 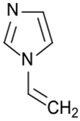	1 W/L AC power; 0.07 mbar; 1.60 mL/min; 5, 9, and 15 min;	RO PA Hydrophilic BW30 TFC (Dow Filmtec Corp.)	Statistically stable compared to its control 44.2	96 to 97 ^4^	NR	Positively charged from pH 3 to 7 and IEP changed from pH 3.5 to ~7	Reduced by 30% from 24 (control) to 17 (15 min)	[8,54]
4	Acrylic acid 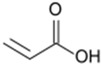	10–50 W RF power; reactor pressure at 0.053, 0.093, and 0.133 mbar; 10–30 min;	MF PP Hydrophobic (Hoechst-Celanese Co.)	Increased by ~50.0% (5 W, 5.332 Pa, and 10 min), compared to its control 48	96.2% of BSA rejection at pH 7 (5 W, 5.332 Pa, and 10 min) ^3^	108 (PP control) declined 25 (5 W, 5.332 Pa, and 30 min)	Negatively charged at pH 7	NR	[12]
5	Acrylic acid	20 W RF power; 25 mL/min (monomer vapor flow rate); 10 min	UF PC(TE) Hydrophobic (Poretics, USA)	NR	NR	Decreased from 71.8 to 36.4 (10 min)	NR	NR	[64]
6	MA with Ar 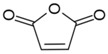	1 W/L AC power; 0.07 mbar; 1.60 mL/min; 5, 9, and 15 min;	RO PA Hydrophilic BW30 TFC (Dow Filmtec Corp.)	Declined by 33% and 18% after 9 and 15 min, compared to its control 44.2	96.8 to 97.5 ^4^	NR	Negative from pH 3 to 8	NR	[54]
7	Triglyme Polyethylene glycol (PEG)—like monomers 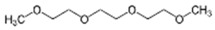	1 W RF power; monomer flow rate: 0.4 sscm at 80–90 °C; 10, 15, 30, 60, and 120 s;	RO PA Hydrophilic SW30HR TFC (Dow Filmtec Corp.)	10–15% decline compared to control, compared to its control 44.2 ^5^	Maintained ~98 ^5^	32 (control) to 7 (modified 120 s)	_	62 (control) to 89 (modified 60 s)	[65]
8	HMDSO, TMMOS, and MTMOS with Ar	30 W RF power; Ar flow rate 10 sscm; 1.5 mbar; 0–20 min;	SiO_2_–ZrO_2_ intermediate layer Laboratory synthesized	High H_2_ permeance of 6.5 × 10^7^ mol/(m^2^ s Pa) with an H_2_/SF_6_ selectivity of 410 at 200 °C	NR	NR	NR	NR	[66]
9	Heptane (C_7_F_16_) and Ar	30, 50, 70 W RF power; 0.03 mbar; monomer flow rate: 5 sccm; heated at 30 °C; 0.03 mbar; 30, 60, 90 s;	PFSA used in proton exchange membrane fuel cell (PEMFC)	Methanol permeability: decreased from 2.42 to 0.033 (10^−6^ cm^2^/s) ^6^	NR	86.9 increased to 117.3 (70 W, 90 s, 400 mTorr)	NR	11.8 increased to 80.2 (70 W, 90 s, 400 mTorr)	[72]
10	Perfluorohexane (C_6_F_14_) and Ar	0.018–0.064 W, 75 kHz discharged; 0.13–0.53 mbar; 5 min; distance between the electrodes is 39 mm;	MF PET-TM (0.4 µm, Sterlitech)	Pure water flux: 3.5–3.6 (its control ~3); Apple juice flux: 2.8–2.9 (its control ~2–2.2)	Sugar rejection: 98–100%	Increased from ca. 48 to 105	NR	Decreased from ca.33 to 14 nm as the degree of deposition increased from 30.3 to 102 µg/cm^2^	[74]
11	Tetrafluoromethane (CF_4_)	50–400 W RF power; monomer flow rate = 18 sccm (standard cm^3^/min); 1–60 min;	UF PES Hydrophilic (Nanjing, China Altrateck Co., Ltd.)	66.7 (control is not given)	100%	Increased from 60 to 125 (modified at 200 W for 40 min)	NR	NR	[75]

^1^ 2000 ppm NaCl solution flowed at a rate of 240 mL/min under 30 bar; ^2^ NR: not report; ^3^ 2 h with BSA solution at a concentration of 1 g/L; ^4^ 2000 ppm NaCl solution under 15 bar, 25 °C; ^5^ Dead-end filtration with 200 mL of 30 g NaCl/L, stirring at 600 rpm; ^6^ With 5 M MeOH, 25 °C.

**Table 4:** The plasma parameters used as variable and constant in three experiment series [78].

Experience Series	Variables	Constants			WCA (Dropped from 137°)
Series 1	Duration	RF Power	Argon Plow Rate	Gap Between Pubstrates and Glow	
	0–150, 30 s interval	100 W	10 slm	5 mm	19° after 150 s
Series 2	Argon Flow Rate	Plasma Power	Duration	Gap between Substrates and Glow	
	0–10, 1 slm interval	100 W	150 s	5 mm	22° at 10 slm
Series 3	Gap Between Substrates and Glow	Duration	Plasma Power	Argon Flow Rate	
	5.0, 7.5, 10, and 12.5 mm	150 s	100 W	10 slm	23° at 10 mm

**Table 5:** Elemental composition of films fabricated by AP-PECVD with different working gases. Data extracted from [67], Copyright (2018), with permission from Elsevier.

Control	Working Gas Component	Elemental Composition (%)				Elemental Ratio	
		Si 2p	C 1s	O 1s	N 1s	C/Si	O/Si
HMDSO		-	-	-	-	3.0	0.5
SiO_2_		-	-	-	-	-	2
	Pure Ar	32.3	3.6	64.1	-	0.11	1.98
	O_2_/Ar (5.0 vol.%)	31.9	3.2	64.9	-	0.10	2.04
	N_2_/Ar (5.0 vol.%)	29.3	20.2	46.0	4.5	0.69	1.57

**Table 6:** Material properties and performance of the polymer membranes upon the atmospheric pressure plasma processes including gas and polymerization (continued on the following two pages).

Entry	Plasma Treatment	Plasma Conditions	Application	Membrane Performance	Water Contact Angle (°)	Pore Size/Porosity	Roughness RMS	Ref.
1	Ar Gas	100 W RF powered two-rotating double-pipe type plasma jets; Ar flow rate = 0–10 slm; 0–150 s; gap between substrates and discharge nozzle: 5.0, 7.5, 10, and 12.5 mm	PVDF-HFP Laboratory synthesized Hydrophobic	For DSSC, the electrolyte update rate is 26.9% higher than the pristine PVDF, 10.8 ± 0.8 g/g	137 declined to 21.3 ± 2.1 at 100 W, 10 slm, 5 mm gap, after 150 s	Increased from 0.6 to 0.7 µm; the porosity increased from 73.6 to 86.0%, compared to pristine control	NR ^1^	[78]
2	AA with Ar/O_2_ or He/O_2_ 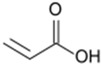	30 kV AC powered plasma jet; Ar or He flow rate: 0.7 m^3^ h^−1^; O_2_ flow rate 0.1 m^3^ h^−1^; 1–20 min; AA heating temperature: 60 °C	MF PP Hydrophobic (Celgard 2500)	As a separator in the lithium-ion battery (LIB), the columbic efficiency maintained at about 99.0% and 99.5% upon 20 min treatment, respectively, compared to the pristine PP (97.5%)	112 (PP control) declined to 63 and 39 upon 20-min Ar/O_2_/AA and He/O_2_/AA, respectively	Increased from 57.8 (control) to 180 nm upon 20-min Ar/O_2_/AA, but decreased to 10 nm upon 20 min He/O_2_/AA	Decreased from 68.91 (control)to 52.73 nm and 46.16 nm after 10 min Ar/O_2_/AA and He/O_2_/AA plasma, respectively	[81]
3	MA with Ar and C_2_H_2_ 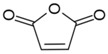	8 W plasma DBD; 5.0–6.6 kHz; 95kPa; MA flow rate: 0.06–0.33; C2H2 flow rate: 2–3 sccm; 5 or 10 min; cap between top and bottom electrodes is 1.6 mm	Silicon (c-Si) wafers	The carboxyl-enriched films were stable when deposited at MA:C_2_H_2_ = 0.037, with a thickness of 544 nm	NR	NR	NR	[83]
4	HMDSO with pure Ar or O_2_/Ar or N_2_/Ar	6 kV DBD plasma jet; 50 kHz; Ar or He flow rate: 0.7 m^3^ h^−1^; O_2_ flow rate 0.1 m^3^ h^−1^; Flow rate of pure Ar or a mixture of Ar with O_2_ (5.0 vol.%) or N_2_ (0.25–10.0 vol.%): 5.0 L min^−1^; 20 min; gap between substrates and discharge nozzle: 2.0 mm; HMDSO heating temperature: 40 °C	Tubular porous α-alumina substrates (SiO_2_-ZrO_2_) Laboratory synthesized	The He permeance of the HMDSO/N_2_/Ar deposited films was 0.52 × 10^−7^, which is lower than that of 1.50 × 10^−7^ mol m^−2^ s^−1^ Pa^−1^, achieved by HMDSO/O_2_/Ar prepared films; HMDSO/N_2_/Ar films also provided highest permeance ratio of He/H_2_ as 1.6	NR	NR	NR	[67]
5	TEGDME with He 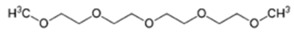	8–13 W AC powered plasma DBD; 15–50 kHz frequency; the total flow of TEGDME/He aerosol and He carrier gas: 8–10 slm; flow rate of He via aerosol: 3.15 slm; 5 min; 4 mm interelectrode gap	Glass substrates	NR	Static WCA is 52 for the film deposited at 27 kHz and 57 at 36 kHz; static WCA of control is not given	NR	NR	[95]
6	Oleate-capped ZnO NPs in *n*-octane (0.5–5 wt.%.)	0.28 ± 0.02 W cm^−2^ AC powered plasma DBD; 10^5^ Pa; total flow of He: 8000 sccm; flow rate of He via aerosol: 2800 sccm; 10 min; 4 mm interelectrode gap	Borosilicate glass slides, CaF_2_ substrates, carbon-coated Cu grids for TEM	NR	Advancing WCA and receding WCA increased from 113 to 170 and from 64 to 168, respectively	NR	The roughness of the films prepared from pure *n*-octane and 3 wt.% NPs dispersion in *n*-octane was 0.345 ± 0.007, and 574 ± 11 nm, respectively.	[93]

^1^ NR: not report.

## Nomenclature

A	atoms
AA-APP	aerosol-assisted atmospheric plasma polymerization
AAc	acrylic acid
AC	alternating current
AlCeO_3_	aluminum–cerium oxide
APP	atmospheric plasma polymerization
AP-PECVD	atmospheric-pressure plasma enhanced chemical vapor deposition
Ar	argon
BSA	bovine serum albumin
C=O	carbonyl group
C_6_F_14_	perfluorohexane
C_7_F_16_	heptane
CF_4_	tetrafluoromethane
CF_x_	fluorocarbon
CO_2_	carbon dioxide
–COO–/–COOH	carboxylic group
CW	continuous wave
DBD	dielectric barrier discharge
DI	deionized
DSSC	dye-sensitized solar cells
e^−^	electrons
FTIR-ATR	Fourier transform infrared spectroscopy-Attenuated total reflectance
H_2_O	water
He	helium
HMDSO	hexamethyldisiloxane
IEP	isoelectric point
LIB	lithium-ion battery
M	monomers
MA	maleic anhydride
MF	microfiltration
MTMOS	methyltrimethoxysilane
N_2_	nitrogen
NC	nanocomposite
NF	nanofiltration
NH_3_	ammonia
NP	nanoparticles
NR	not report
O_2_	oxygen
OES	optical emission spectroscopy
-OH	hydroxyl group
PA	poly(amide)
PC	poly(carbonate)
PECVD	plasma enhanced chemical vapor deposition
PEG	poly(ethylene glycol)
PEO	poly(ethylene oxide)
PES	poly(ethersulfone)
PET	poly(ethylene terephthalate)
PET-TM	poly(ethylene terephthalate) track-etched membranes
PFSA	perfluorosulfonic acid
PP	poly(propylene)
PSf	poly(sulfone)
PTFE	poly(tetrafluoroethylene)
PVDF	poly(vinylidene fluoride)
PVDF-HFP	poly(vinylidene fluoride-co-hexafluoropropylene)
RF	radio frequency
RMS	roughness
RO	reverse osmosis
sccm	standard cubic centimeter per minute
SEM	scanning electron microscope
SiO_2_	silica
SiO_2_–ZrO_2_	silicon dioxide–zirconium dioxide
SiO_x_	silicon oxide
slm	standard liters per minute
TFC	thin film composite
TMMOS	trimethylmethoxysilane
TSEM	transmission scanning electron microscopy
UF	ultrafiltration
VIM	1-vinylimidazole
WCA	water contact angle
XPS	X-ray photoelectron spectroscopy
ZnO	zinc oxide
